# The Occurrence of Acute Disseminated Encephalomyelitis in SARS-CoV-2 Infection/Vaccination: Our Experience and a Systematic Review of the Literature

**DOI:** 10.3390/vaccines11071225

**Published:** 2023-07-10

**Authors:** Adina Stoian, Zoltan Bajko, Mircea Stoian, Roxana Adriana Cioflinc, Raluca Niculescu, Emil Marian Arbănași, Eliza Russu, Marian Botoncea, Rodica Bălașa

**Affiliations:** 1 Department of Pathophysiology, George Emil Palade University of Medicine, Pharmacy, Science and Technology of Targu Mures, 540139 Targu Mures, Romania; adina.stoian@umfst.ro (A.S.); raluca.niculescu@umfst.ro (R.N.); 2 Department of Neurology, George Emil Palade University of Medicine, Pharmacy, Science and Technology of Targu Mures, 540139 Targu Mures, Romania; zoltan.bajko@umfst.ro (Z.B.); rodica.balasa@umfst.ro (R.B.); 3 Department Anesthesiology and Critical Care Medicine, George Emil Palade University of Medicine, Pharmacy, Science and Technology of Targu Mures, 540139 Targu Mures, Romania; mircea.stoian@umfst.ro; 4 1st Neurology Clinic, Mures County Emergency Hospital, 540136 Targu Mures, Romania; roxana.cioflinc@yahoo.ro; 5 Doctoral School of Medicine and Pharmacy, George Emil Palade University of Medicine, Pharmacy, Sciences and Technology of Targu Mures, 540142 Targu Mures, Romania; emilarbanasi1@gmail.com; 6 Department of Vascular Surgery, George Emil Palade University of Medicine, Pharmacy, Science and Technology of Targu Mures, 540139 Targu Mures, Romania; 7 Clinic of Vascular Surgery, Mures County Emergency Hospital, 540136 Targu Mures, Romania; 8 Department of General Surgery, George Emil Palade University of Medicine, Pharmacy, Science and Technology of Targu Mures, 540139 Targu Mures, Romania; marian.botoncea@umfst.ro

**Keywords:** acute disseminated encephalomyelitis, acute hemorrhagic leukoencephalitis, SARS-CoV-2 vaccine, COVID-19, ADEM, vaccination, demyelinating disease of CNS, autoimmune disease, coagulopathy

## Abstract

Background: The neurological complications of coronavirus disease 2019 (COVID-19) infection and the side effects of vaccination include immune-mediated diseases of the central nervous system (CNS) such as acute disseminated encephalomyelitis (ADEM). It is an acute-onset demyelinating disease that involves a rapid evolution and multifocal neurological deficits that develops following a viral or bacterial infection or, less frequently, following vaccination. Acute hemorrhagic leukoencephalitis (AHLE) is the hemorrhagic variant of ADEM that presents a more severe evolution which can be followed by coma and death. The objectives of this study consist in evaluating the diagnosis, clinical characteristics, imaging and laboratory features, evolution, and treatment of ADEM and AHLE following COVID-19 infection or vaccination. Methods: We performed a systematic review of the medical literature according to PRISMA guidelines that included ADEM cases published between 1 January 2020 and 30 November 2022 following severe acute respiratory syndrome coronavirus 2 (SARS-CoV-2) infection and vaccination and also included our own clinical experience regarding this pathology. Results: A total number of 74 patients were diagnosed with ADEM, 45 following COVID-19 infection and 29 after a SARS-CoV-2 vaccine. A total of 13 patients (17.33%) presented AHLE. The moderate form of COVID-19 presented a positive correlation with AHLE (r = 0.691, *p* < 0.001). The existence of coma and AHLE was correlated with poor outcomes. The following more aggressive immunomodulatory therapies applied in severe cases were correlated with poor outcomes (major sequelae and death): therapeutic plasma exchange (TPE) treatment (r = 382, *p* = 0.01) and combined therapy with corticosteroids and TPE (r = 0.337, *p* = 0.03). Conclusions: Vaccinations are essential to reduce the spread of the COVID-19 pandemic, and the monitoring of adverse events is an important part of the strategic fight against SARS-CoV-2. The general benefits and the overall good evolution outweigh the risks, and prompt diagnosis is associated with a better prognosis in these patients.

## 1. Introduction

Coronaviruses are well-known pathogens that affect humans and animals, being responsible for respiratory tract infections. In 2019, a mutated form of a coronavirus, which resulted in a worldwide pandemic considered as the most tragic in our history, was first identified in Wuhan, China. Respiratory symptoms were initially identified as prominent, but soon after, the involvement of different systems and organs was reported following coronavirus disease 2019 (COVID-19) infection, including neurological complications [1]. The most common neurological complications of severe acute respiratory syndrome coronavirus 2 (SARS-CoV-2) infection include immune-mediated diseases; encephalopathy and encephalomyelitis; ischemic stroke; neuromuscular disorders; and smell and taste disorders [1,2]. Some of these complications—such as neuromuscular diseases, Guillain–Barre syndrome (GBS), stroke, and acute disseminated encephalomyelitis (ADEM)—appear at the onset or during active infection, while others appear after a latent period; these include autoimmune encephalitis and post-COVID-19 neurological syndrome (characterized by brain frog and cognitive dysfunction; sleep disorders; mood disorders; and smell and taste disorders) [3].

ADEM is an acute-onset demyelinating disease of the central nervous system (CNS) that affects the cerebral hemispheres, cerebellum, brainstem, or spinal cord with rapidly evolutive, multifocal neurological deficits, and usually evolves in a monophasic way and develops following a viral or bacterial infection (usually involving the upper respiratory tract) or less frequently following vaccination [4,5,6,7].

Case reports of a rising number of patients with ADEM following viral infection or SARS-CoV-2 vaccination have been published.

The objectives of this study consist in providing an overview on the diagnosis, clinical characteristics, imaging and laboratory features, evolution, and treatment of ADEM following COVID-19 infection or vaccination. We also wanted to evaluate if acute hemorrhagic leukoencephalitis (AHLE) appears more frequently postvaccination or postinfection and if there are evolutionary differences compared with the classic form.

### 1.1. General Incidence of ADEM

Children and adolescents are most commonly affected, but cases have also been reported in adults and elderly patients [8]. The incidence of ADEM in childhood is ~0.5/100,000 patients [9]. The annual global incidence of ADEM is 1 in 125,000–250,000 individuals per year, and according to some reports it seems to be more common in males than in females [10]. Other studies also revealed a similar global incidence, estimated at 0.8/100,000 [8,11].

### 1.2. Etiology

The appearance of ADEM is considered secondary to viral exposure, or less often (in 5% of cases) following vaccination [12,13]. Infections by various pathogens have been reported to result in ADEM—especially herpes simplex, coronaviruses, influenza, Epstein–Barr virus, cytomegalovirus, and measles [14,15]. Many vaccines have been associated over time with side effects like GBS and transverse myelitis. In young people, narcolepsy was reported after they received the influenza vaccine [16,17]. Postvaccinal ADEM is described especially after influenza, varicella, measles, mumps, rabies, hepatitis B, diphtheria, and tetanus immunization [5,18]. Isolated case reports and case series in the current context of the last 3 years show a relationship between SARS-CoV-2 virus and ADEM, with both postviral and postvaccinal cases being described, secondary to the efforts made to combat the pandemic [12,13]. Until now, there have been no large population studies to evaluate the incidence of these cases. 

## 2. Materials and Methods

We performed a systematic review of the medical literature according to PRISMA guidelines (Preferred Reporting Items for Systematic Review and Meta-Analyses) (http://www.prisma-statement.org/, accessed on 30 November 2022) using articles available in the PubMed database, and a predefined combination of search terms: “acute disseminated encephalomyelitis” or “ADEM” and “COVID-19” or “SARS-CoV-2” or “SARS-CoV-2 vaccine” or “COVID-19 vaccine”. The literature research was performed by 2 independent reviewers (AS and RC) and all articles with relevant titles published between 1 January 2020 and 30 November 2022 were subjected to a systematic analysis and included in the review if the content was relevant to the current study. An evaluation was solicited from a third reviewer (MS) if there were discrepancies or doubts regarding the relevance of some articles.

Inclusion criteria: age of the reported patient(s) was over 18 years; confirmed ADEM diagnosis after COVID-19 or administered SARS-CoV-2 vaccine; magnetic resonance imaging (MRI) performed; presence of relevant information regarding the collected data; and a clear description of the cases. Only papers in English were considered.

Exclusion criteria: duplicate articles; reports published only as abstracts; reports published in a language other than English; studies that contained insufficient data; general reviews; and neurologic disease other than ADEM.

**Figure 1:**
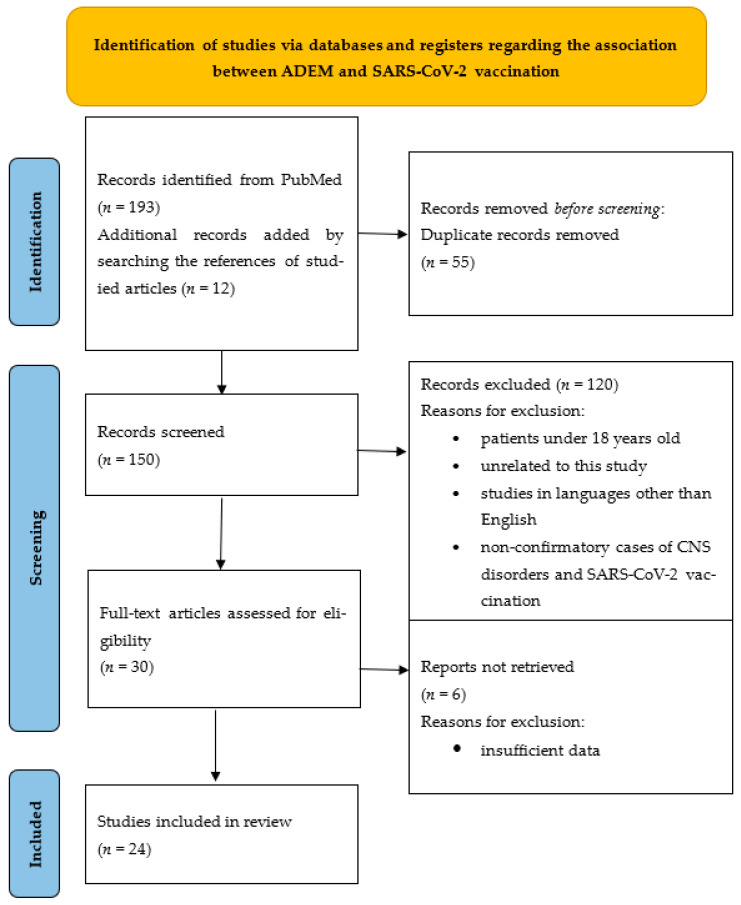
Identification of studies via databases and registers regarding the association between ADEM and SARS-CoV-2 vaccination.

For the association between vaccination and ADEM, a total of 193 articles met the criteria using our defined keyword search. The number of articles increased by 12 after we screened and analyzed the reference lists of found articles and discovered additional case series and case reports. Duplicate records were removed (*n* = 55) and 150 articles were screened and analyzed. There were 120 publications that were eliminated because they were written in a language other than English, they did not include information on COVID-19 infection or immunization status, or the patients were children. In addition, 6 studies were excluded due to inadequate data for our analysis. Finally, 24 publications fulfilled the inclusion and exclusion criteria and were included in the review. From these, we identified 28 patients who developed postvaccinal ADEM. We added our own case to the total number of patients when we performed the statistics. The flow chart of the research strategy is illustrated in Figure 1. The following data were extracted from the selected articles: age, gender, type of administered vaccine, reverse transcription-polymerase chain reaction (RT-PCR) test swab (performed/not performed), the onset latency for neurological symptoms after vaccine, neurological symptoms, brain and spine MRI, cerebrospinal fluid (CSF) analysis, other lab tests carried out, treatment, and outcome.

**Figure 2:**
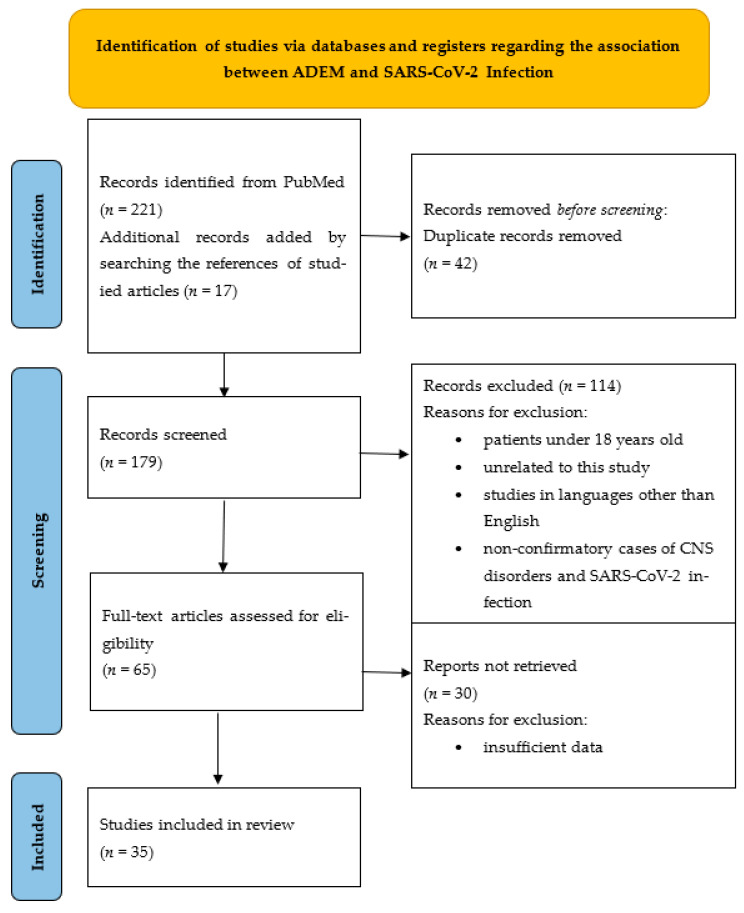
Identification of studies via databases and registers regarding the association between ADEM and SARS-CoV-2 infection.

For the association between infection and ADEM, a total of 221 articles met the criteria according to the searched keywords. Another 17 articles with additional case series and case reports were discovered after we screened and analyzed reference lists. Duplicate records were removed (*n* = 42) and 179 articles were screened and analyzed. A total of 114 articles were eliminated because they were written in a language other than English, the patients had no history of COVID-19 infection, or the patients were under the age of 18. Another 30 research articles were also eliminated because of inadequate data for our study. In the end, 35 publications fulfilled the inclusion and exclusion criteria and were finally included in the review. We identified 45 patients reported in the included articles that developed postinfectious ADEM. The flow chart of the research strategy is illustrated in Figure 2. The following data were extracted from the selected articles: age, gender, RT-PCR test swab (performed/not performed), the onset latency for neurological symptoms after infection, neurological symptoms, brain and spine MRI, CSF analysis, other lab tests carried out, treatment, and outcome. Data from all the articles and the characteristics of the patients included in the review are compiled in two tables (Table 1 and Table 2).

## 3. Results

### 3.1. Our Clinical Experience

A previously healthy, 33-year-old male presented at the emergency room and was admitted to the neurology clinic in July 2021 with a 3-day history of fever, headache, nausea and vomiting, decreased muscle strength of the limbs with a predominance in the lower limbs, paresthesia, and urinary difficulties with urinary retention—symptoms that started 14 days after receiving his first dose of the Johnson & Johnson vaccine.

On neurological examination, he was aware and fully alert to the place, time, and person; negative for nuchal rigidity; and had cranial nerves in normal limits. The examination of muscle strength as assessed by Medical Research Council (MRC) grading revealed spastic tetraparesis, grade 4+/5, in the upper limbs and 4/5 in the lower limbs; increased deep tendon reflexes in the lower limbs; bilateral Babinski signs; and acute urinary retention. He had no significant personal history of previous diseases and had no family history suggestive of autoimmune disease, but he was overweight. He had no contact with SARS-CoV-2-positive cases and the RT-PCR swab test was negative.

Upon admission, laboratory tests revealed the following: white blood cells (WBC): 14,200/mm^3^; red blood cells (RBC): 5.31 × 10^6^/µL; hemoglobin: 15.5 g/dL; hematocrit: 46.3%; platelets (PLT): 371.000/mm^3^; a blood sugar level of 154 mg% (repeated fasting glycemia: 120, 130 mg%); D-dimers: 279 ng/mL; C-reactive protein (CRP): negative; fibrinogen 301 mg/dL; cholesterol: 244 mg/dL; triglycerides: 126 mg/dL; alanine transaminase (ALT): 22 IU/L; aspartate aminotransferase (AST): 46 IU/L; blood urea nitrogen (BUN): 30 mg/dL; creatinine: 1.03 mg/dL; glomerular filtration rate (GFR): 102 mL/min; sodium (Na): 145 mmol/L; potassium (K): 4.17 mmol/L; magnesium (Mg): 0.95 mmol/L; total serum calcium (Ca): 2.43 mmol/L; total proteins: 6.84 g/dL; and anticardiolipin antibodies: 1.3 IU/mL.

Other blood tests, such as microbiologic and serological tests, including a large panel for neurotropic viruses, were performed (cytomegalovirus, Epstein–Barr virus, human immunodeficiency virus [HIV] 1 and 2, herpes simplex viruses 1 and 2, hepatitis B and C, varicella zoster virus, rubella, hepatitis), and all were within normal values.

Treponema pallidum, toxoplasmosis, and Borrelia burgdorferi were negative and ruled out. Blood tests for endocrinopathies, autoimmune diseases (neuromyelitis optica [NMO] antibodies [anti-aquaporin-4], anti-double-stranded DNA [anti-dsDNA] antibodies, antinuclear antibodies, anti-myelin oligodendrocyte [MOG] antibodies, and an autoimmune panel for encephalitis) were also performed and all the results were negative. The screening for neuronal antibodies was also negative.

The CSF had normal pressure and was clear and colorless. The CSF analyses revealed the following values: glucose: 93 mg/dL; proteins: 469 mg/dL; RBC: 0/uL; and WBC: 650/uL (95% lymphocytes, but no lymphoid cells suggestive of lymphoma were present in the CSF). The CSF examination did not reveal other abnormalities or bacterial, fungal, or Mycobacterium tuberculosis infection; and an immunoelectrophoretic exam of serum and CSF revealed no oligoclonal bands. Serology for SARS-CoV-2 in CSF was not performed. The patient underwent a thoracoabdominal–pelvic computed tomography scan that was within normal limits, ruling out neoplasia.

**Figure 3:**
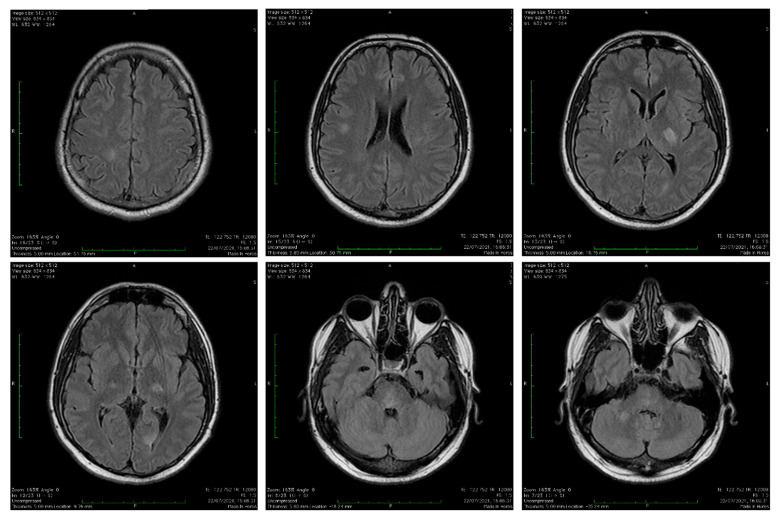
Axial cerebral MRI sequences. Multiple hyperintense lesions in the cerebral hemispheres, brainstem, and cerebellum.

Brain MRI 1,5 tesla (T) including gadolinium contrast administration revealed multifocal T2 and T2 fluid-attenuated inversion recovery (FLAIR), and showed multiple T2/FLAIR hyperintense, poorly demarcated lesions that involved the white matter, right frontal and parietal lobes, left occipital lobe, left basal ganglia, pons, and right cerebellar peduncle, without contrast enhancement (Figure 3).

**Figure 4:**
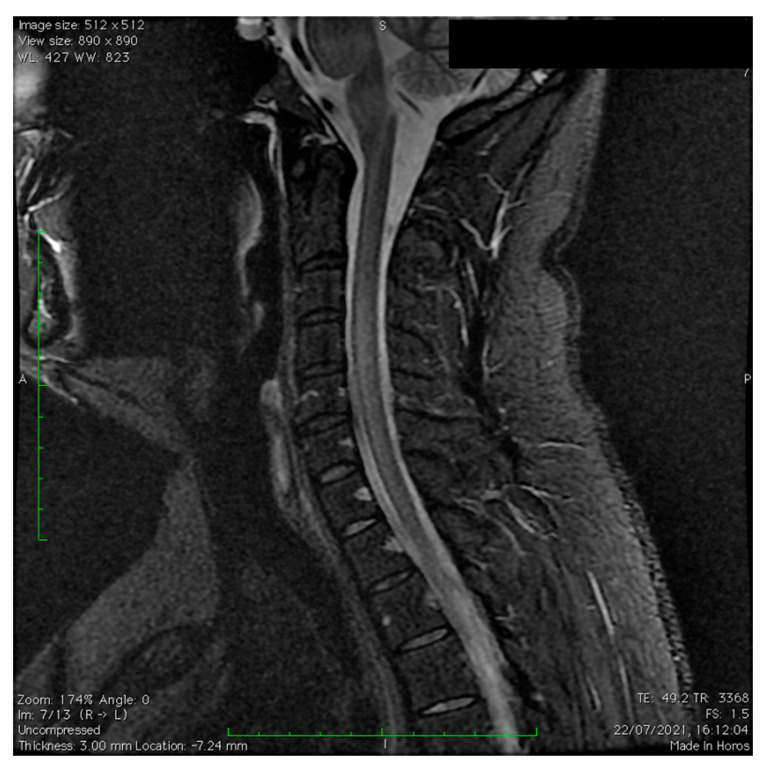
Sagittal spinal cord MRI, STIR sequences. Longitudinally extensive lesion at the level of the cervical spinal cord.

The spine MRI revealed hyperintense areas in T2 and FLAIR images that occurred in the cervical region at C2, C4-C5, and C7, without contrast enhancement (Figure 4).

Empiric therapy with antibiotics and acyclovir was started first. Based on these clinical, imaging, and CSF results, and taking into account the temporal relationship with the administration of the Johnson & Johnson vaccine, we concluded that the diagnosis was ADEM postviral vector vaccination and continued with intravenous methylprednisolone (IV MP). The patient received 1 gr of IV MP for 5 days with marked improvement in the symptomatology, and he was discharged on oral steroids with a tapering regime. A repeated brain MRI on day 40 following the initial examination showed a significant reduction in the diameter of the demyelinating lesions, without any new lesions.

Our patient met the diagnostic criteria for ADEM according to the International Pediatric Multiple Sclerosis Study Group 2007, and the correlation between neurological clinical presentations, lab tests, and MRI findings led to the diagnosis of ADEM. An association with the vaccine was suspected based on the temporal relationship between vaccine administration and the onset of neurologic disease. After pathogen-induced encephalitis was excluded, the presence of pleocytosis and the absence of intrathecal oligoclonal band synthesis pointed to the diagnosis of ADEM. Although the connection between the vaccine and the neurological disease may be coincidental, there is still the possibility of a secondary neuroinflammatory syndrome.

The positive diagnosis is supported by the following:

(1) The temporal association between the infection/vaccine and disease;
(2) Clinical features;
(3) Appearance on MRI images;
(4) Exclusion of other etiologies;
(5) Favorable response to corticosteroids.

Informed consent was obtained from the patient for the publication of his data and his accompanying MRI images. To the best of our knowledge, this is the first case of postvaccinal ADEM reported in Romania in the context of the COVID-19 pandemic.

### 3.2. Literature Review

A total number of 74 patients were diagnosed with ADEM, 45 patients (60.81%) after COVID-19 infection and 29 (39.19%) after a SARS-CoV-2 vaccine. A total of 13 patients (17.33%) out of the total number of patients presented AHLE (22.22% after infection and 10% after vaccine), without a statistically significant difference (*p* = 0.18).

The average time between infection with SARS-CoV-2 and the onset of ADEM was 19.53 days, and the average time between the vaccine administration and the onset of ADEM was 12.34 days (*p* = 0.04). In the postinfectious group, a statistically significant correlation was not found between the severity of COVID-19 and the outcome; however, there was a positive correlation between the moderate form of COVID-19 and AHLE (r = 0.691, *p* < 0.001). Brain MRIs were performed on 73 patients (98.64%) and spine MRIs on 21 patients (28.37%). Contrast enhancement was reported in 27 cases (36.48%), without a significant difference between the groups.

Oligoclonal bands (OCB) were present in 12 cases (16%), and anti-MOG antibodies were present in 1 case. Moreover, SARS-CoV-2 RT-PCR from CSF was positive in four patients (8.89%) from the postinfectious group, with the precise result that only two patients were positive at the time of the PCR swab test.

The most frequent immunosuppressive therapy administered was corticosteroid therapy (87.83%), alone or in combination with other therapies, followed by IVIg (32.43%), plasmapheresis (17.56%), and rituximab (5.40%). No statistically significant relationship was observed between the administered therapy and the clinical evolution of the disease, with all the administered classes seeming to have similar clinical efficiency. Regarding the combined treatment, in the postvaccination group we see a higher incidence of corticosteroid and plasmapheresis therapy (*p* = 0.04). The rest of the combined therapies and their incidences are presented in Table 3.

In terms of the therapy used (Table 4) and clinical outcome, for the Spearman correlation we have a positive correlation between monotherapy (regardless the administrated therapy), corticosteroid monotherapy, and full recovery (r = 0.352, *p* = 0.003; and r = 0.384, *p* = 0.001) for all patients, as well as for postvaccination ADEM patients (r = 0.493, *p* = 0.007; and r = 0.529, *p* = 0.003), but not for the postinfection COVID-19 patients.

In contrast, in terms of poor outcomes (major sequelae and death), as seen in Table 5, we have a positive correlation between the TPE treatment (r = 0.382, *p* = 0.01), combined therapy with corticosteroids and TPE (r = 0.337, *p* = 0.03), and the abovementioned endpoint in all patients. Moreover, the presence of coma (r = 0.501, *p* < 0.001) and AHLE (r = 0.314, *p* = 0.006) is correlated with poor outcomes. For the postinfection group, TPE therapy (r = 0.314, *p* = 0.04) and coma (r = 0.389, *p* = 0.008) presented a positive correlation with poor outcomes. Furthermore, for the postvaccination patients, AHLE (r = 0.665, *p* < 0.001) and coma (r = 0.449, *p* = 0.01) were associated with poor outcomes.

## 4. Discussion

### 4.1. Pathophysiology of ADEM

The relationship between infection/vaccination and the occurrence of demyelinating diseases is not fully understood, being attributed to an exaggerated autoimmune reaction of the body to viral or vaccine antigens [73].

Experimental autoimmune encephalomyelitis in animal models can be triggered after immunization with CNS homogenate with myelin peptides emulsified in complete Freud’s adjuvant, and this is used to study the mechanisms underlying ADEM with inflammatory demyelinating lesions in the brains and spinal cords of experimental animals [8,74].

Additionally, Theiler proposed in the 1930s a murine encephalomyelitis model as a model to study the pathogenic infectious mechanisms of the disease, consisting in inoculation of susceptible mouse strains in the cerebral hemisphere with the Theiler murine encephalomyelitis virus. The disease seems to be triggered by cluster of differentiation (CD)8+ T cells, while ongoing inflammation is sustained by CD4+ T cells which infiltrate the CNS and recruit additional mononuclear cells and lymphocytes to cross the blood–brain barrier (BBB), finally producing inflammation and demyelination [8,75].

Based on animal model research, two theories have been developed:

(a) The concept of molecular mimicry is based on the similarity of an amino-acid sequence (epitope) between myelin proteins of the host and invading pathogens [75,76]. The antigen-presenting cells (dendritic cells) process the pathogen, activating T cells which in turn activate B cells. Both of these cell types are able to enter into the central compartment during the process of immune surveillance and can be reactivated by local antigen-presenting cells (microglia), producing a local inflammatory immune reaction [8,75]. The injection of CD4+ T lymphocytes from immunized animals that recognize myelin-associated protein can initiate the disease in healthy animals [75,77].
(b) CNS infection with a pathogen results in nervous tissue damage with the penetration of autoantigens in systemic circulation through a disrupted BBB. These autoantigens reach the lymphatic organs, where they are processed and initiate a self-reactive T-cell response with nonspecific activation of an autoreactive T-cell clone [8,75].

The proposed postinfectious and postvaccinal mechanisms are molecular mimicry, bystander activation, epitope spreading, and polyclonal B-cell activation [3,78]. In the context of inflammation that produces increased vascular permeability in the CNS, molecular mimicry between viral proteins and myelin antigens is followed by a cross-reaction driven by a T-cell-mediated autoimmune response directed against myelin basic protein [32,34]. Talbot et al. reported human coronavirus myelin–T-cell cross-reactivity in patients with multiple sclerosis (MS) [79]. Postvaccinal ADEM generally appears after between 1 and 14 days, especially after the first dose of the vaccine, and rarely after revaccination [34,80].

Postinfectious ADEM is characterized from a morphopathological point of view by the existence of perivenous demyelinating lesions, lymphocytic and macrocytic infiltrates along with endothelial swelling, perivascular edema, and hemorrhages, followed in late stages by foci of fibrillary fibrosis [75].

### 4.2. Pathophysiology of ADEM after SARS-CoV-2 Infection

Currently, it is clear that SARS-CoV-2 is a neurotrophic virus and that the neurological damage directly involves viral lesions of the glial cells and neurons, neuroinflammation, hypercoagulability, and endothelial dysfunction [49,81]. SARS-CoV-2 penetrates the CNS, producing neurological complications. The most common methods of penetration mentioned are the following:

(1) Systemic circulation can contribute to the distribution of the virus in the cerebral blood flow and from here, due to sluggish blood flow in the context of inflammation, viral neuroinvasion is facilitated [82].
(2) The virus crosses the BBB due to increased permeability in the context of a cytokine storm [9].
(3) The virus is carried by the infected immune cells—leukocytes [9]—that function as a viral reservoir and can infiltrate the brain tissue through the glymphatic (glial–lymphatic) system—the so-called Trojan horse mechanism [1,82,83].
(4) The spike protein of the virus binds to cell-surface angiotensin-converting enzyme type 2 (ACE2) receptors found in various tissues and infects the endothelial cells of the BBB or the epithelial cells of the blood–CSF barrier at the level of the choroid plexus—mediating cellular entry of the virus towards the central compartment (brain and brainstem—the nucleus of the solitary tract and the paraventricular nuclei) [1,35,83].
(5) Penetration occurs via a neuronal route by retrograde axonal transport from the peripheral nerves towards the CNS through synaptic connections (olfactory nerves) [1,84].

Viral agents, including SARS-CoV-2, can disrupt the immunomodulatory mechanism due to a transient immunosuppression in the periphery with lymphopenia and an aberrant immune reconstitution that leads to perturbed immunoregulation, breakdown of self-tolerance, and reactivation of self-reactive lymphocytes even in the absence of epitopes common to self-antigens [77]. The generation of a systemic inflammatory response (SIRS) causes the excessive production of proinflammatory cytokines (interleukins: IL-6, IL-12, IL-15, TNF-α), resulting in a cytokine storm that also affects the CNS. The BBB is compromised by the cytokine storm with increased permeability, which triggers an innate local immune response in resident cells with activation of glial cells in the CNS compartment, and infiltration of cytotoxic T lymphocytes in brain parenchyma. This induces a powerful proinflammatory state and initiates autoimmunity [1,84]. A recent study found a compartmentalized response when analyzing the blood and the CSF of patients with COVID-19 with CNS-specific T-cell and B-cell activation and antineuronal reactivity [85].

Neurological disease can occur early in the evolution of COVID-19 as the result of virus invasion, which could explain, in some cases, the early onset of neurological symptoms after diagnosis with COVID-19. Alternatively, it can occur in the recovery phase through a postinfectious, immune-mediated mechanism: the virus induces an autoimmune reaction after a latent period following acute infection. This is explained by the hypothesis of molecular mimicry between viral and self-antigens [83]. Not much is known about the immune-mediated diseases of the CNS secondary to SARS-CoV-2 infection. In most cases with neurological involvement in the context of COVID-19, the presence of the virus in CSF was not highlighted, probably due to reduced viremia or a rapid viral clearance, and there were only isolated cases that reported a RT-PCR test of CSF [3].

Generally, vaccines stimulate a strong pathogenic response from T cells, with an increase in the level of proinflammatory cytokines—as demonstrated in the case of the ChAdOx1 nCoV-19 vaccine [86,87]. The antigens contained in the vaccine are recognized as potential pathogens by the peripheral circulating immune cells (macrophages and monocytes) and induce the transcription of the target genes with increased synthesis of inflammatory and pyrogenic cytokines (IL-1, IL-6, tumor necrosis factor [TNF] α). These enter into the bloodstream, creating a response that is similar to infection. Phagocytosis is then initiated and stimulated in the immune system with further release of inflammatory mediators—including cytokines, chemokines, activation of the complement system, and cellular recruitment. Inflammatory mediators released into the circulation can induce systemic side effects including microglia activation and neuroinflammation, depending on the immunogenetic background [86,88,89]. Several pathogenic mechanisms, like molecular mimicry, aberrant immune responses with immune cell activation and infiltration, maladaptive immune responses, an inflammatory cascade, and direct neurotoxicity, have been used to explain the association between vaccines and neurological manifestations [16,17]. In molecular mimicry, systemic or intrathecal antibody synthesis against some myelin proteins (myelin basic protein, myelin oligodendrocyte glycoprotein, and proteolipid protein) with which the virus shares antigenic properties leads to a cross-reaction of the antibodies produced by infection or following vaccination [15]. The molecular analyses of anti-SARS-CoV-2 antibodies demonstrated a cross-reaction of antibodies directed against the viral spike protein with some human antigens, including neurofilament proteins [90]. The autoimmune reaction and increased central system blood vessel permeability can explain the favorable effect of anti-inflammatory therapy [49,91,92].

### 4.3. ADEM Diagnosis

Diagnostic criteria were developed for the pediatric population by the International Pediatric Multiple Sclerosis Society Group in 2007 and were updated in 2013. However, there are no clearly defined criteria for the adult population [27].

(1) Multifocal damage of CNS at first manifestation due to an inflammatory demyelinating cause.
(2) Encephalopathy that cannot be explained by a rise in fever.
(3) Lack of other clinical events or new lesions on MRI in the 3 months following onset.
(4) Brain and/or spine MRI shows lesions in the acute phase (3 months).
(5) Brain lesions on MRI are diffuse and poorly demarcated and have the following characteristics:

(a) Large-size lesions of 1–2 cm that mainly affect the white matter.
(b) Hypointense T1 lesions affecting white matter are rare.
(c) Lesions may also be present in deep gray matter [27,93].

### 4.4. General Considerations on Postinfectious and Postvaccinal ADEM in the Context of COVID-19

From December 2020, vaccination started being approved worldwide as a safe solution designed to protect individuals from the virus and to prevent progression to the severe form of the disease [20,94]. Although studies carried out so far indicate that the vaccines against SARS-CoV-2 have a high safety profile, and none of the currently approved vaccines use live attenuated viruses, postvaccination neurological complications including ADEM have nevertheless been reported [53]. The full spectrum of complications for these vaccines is not yet fully known.

Current vaccines used against COVID-19 include the following:

(1) mRNA-based vaccines in which human cells are stimulated to produce SARS-CoV-2 proteins and express the viral spike protein on their surface by means of genetically transferred information. The human body then initiates a defensive response against it.
(2) Viral vector-based vaccines in which an adenovirus is used to deliver fragments of the SARS-CoV-2 genome to human cells.
(3) Inactivated viral vaccines in which a dead SARS-CoV-2 virus triggers the immune response after inoculation [16,17].

The vaccine developed by Johnson & Johnson (COVID-19 Vaccine Janssen) uses a nonreplicating viral vector to deliver a fragment of SARS-CoV-2 genetic information to host cells. This genetic information is necessary for the synthesis of the SARS-CoV-2 spike protein that subsequently acts as an antigenic protein. The viral vector used is an adenovirus without replicative capacity, which is considered safe for immunocompromised patients [53].

ChAdOx1n COV-19 contains an adenoviral vector that encodes the spike protein of SARS-CoV-2 [95]. Both vector-based vaccines and mRNA vaccines encode and stimulate the production of the SARS-CoV-2 spike protein [33]. Messenger RNA is recognized by cytosolic and endosomal toll-like receptors (TLR3, TLR7), while the vector-based vaccines contain elements of the virus particle that are recognized by pattern recognition receptors (TLR9) [33]. The ChAdOx vaccine elicits a strong T-cell response based on a Th1-phenotype [96]. Infection and vaccination trigger a strong immune response with increased expression of T lymphocytes and proinflammatory cytokines. Viral or vaccine antigens are recognized by peripheral circulating immune cells (monocytes, macrophages) through surface receptors, resulting in an increase in the expression of many target genes, increased synthesis of inflammatory cytokines, complement activation, and phagocytosis initiation with further cell recruitment [86,87].

The neutralizing antibodies compete with ACE2 for the receptor-binding domain of SARS-CoV-2, and it is suggested that postinfection and postvaccinal antibodies could show an aberrant affinity for endogenous ACE2-receptors, increasing the risk of autoimmune reactions in the areas of the brain rich in ACE2 receptors (periventricular lesions) [35,97,98]. This would also explain the impaired function of ACE2 receptor-rich endothelial cells belonging to the cerebral microvasculature that leads to increased BBB permeability with demyelination, like in ADEM [35].

Additional factors belonging to the host, like cell-surface proteins including neutrophilin-1 (very well expressed in the olfactory nerve and human brain with an important role in endothelial function, neuronal development, and modulation of innate immune responses), are presumed to facilitate virus entry into cells [35,99,100]. The alteration of neutrophilin-1 expression correlates with endothelial and BBB dysfunctions, neuroinflammation from experimental autoimmune encephalomyelitis, and the severity of immune responses produced by COVID-19 [35,101,102].

The incidence of ADEM after SAR-CoV2 vaccines has not yet been reported worldwide, but some data from India show an incidence of 3 cases/8.19 million ChAdOx1 vaccines, so without a statistically significant increase, this might raise questions about the safety of these vaccines [30]. Messenger RNA vaccines are a new type of vaccine, but as of 26 May 2021, 9442 adverse reactions were reported to the Vaccines Event Reporting System (VAERS) database, including some rare neurological complications and six cases of ADEM [103]. Until the end of March 2022, more than 170 patients with postvaccinal ADEM were reported to the Eudra Vigilence database of the European Medicine Agency (91 patients after the BioNTech Pfizer Vaccine, 46 after the AstraZeneca vaccine, 27 after the Moderna vaccine, and 8 following the Johnson & Johnson vaccine) [33]. Different institutions collect data regarding adverse reactions that occur after administration of COVID-19 vaccines. For example, the National Institute of Public Health of Quebec (INSPQ) reported 67 side effects in Quebec for each 100.000 doses administered, for all types of vaccines. The proportion is higher for AstraZeneca, with 182.5 reported cases per 100.000 doses, but most of them were labeled as “without gravity” [86]. The European Medicine Agency (EMA) reported only 10 cases of ADEM between 20/01/21 and 10/06/21, after almost 46 million doses of the CgAdOx1 nCoV-19 vaccine were administered—so its protective effects far outweigh any side effects [1,104]. Cases of ADEM are more frequently reported after SARS-CoV-2 infection than postvaccination [14].

The published literature reveals a constellation of manifestations in patients, suggestive of multifocal CNS involvement. The first case of COVID-19-associated ADEM was reported by Zhang et al. in a 40-year-old woman [105]. Shortly thereafter, more case reports or case series appeared.

In ADEM, pathological findings on MRI are multifocal, bilateral, sometimes with confluent T2/FLAIR hyperintensities, often asymmetrical and bilateral, sometimes tumefactive, and with poorly defined borders on T2-weighted and FLAIR images. The lesions are situated in the cortical peripheral gray matter, subcortical gray matter, and white matter junction but also in the basal ganglia, thalami, brainstem, and cerebellum, with a variable enhancement pattern. Unlike in MS, the corpus callosum is spared [49,73]. MRI findings in ADEM may overlap with MS, but the latter is characterized by periventricular white matter, corpus callosum, and subcortical U fiber involvement [12,106]. Usually, the plaque borders in ADEM are not clearly defined. While in MS the lesions are permanent, in ADEM there is a nonspecific gliosis with no myelin loss, and axons are also generally preserved, which explains the clinical evolution with total recovery in some cases [107]. The involvement of gray matter, the lack of periventricular lesions and of T1-black holes, and the absence of Dawson finger configuration are helpful in distinguishing ADEM from MS [108,109]. A particularity of COVID-19-associated ADEM is the presence of a linear perivascular enhancement that correlates with the changes highlighted in the biopsy with perivenous inflammation and inflammatory infiltrates of lymphocytes and macrophages [49].

Acute hemorrhagic leukoencephalitis (AHLE) is a hyperacute and more severe subtype of ADEM. It is much rarer, with a poor prognosis and a more severe course that can rapidly progress to coma and death. This variant was first described by Hurst in 1941 [110] and can occur at any age, but predominates in children and young adults. AHLE is characterized by the presence of T2 and FLAIR hyperintense lesions and edema in the deep white matter and subcortical areas, as well as T1 hypointense lesions with microhemorrhages in susceptibility-weight images (SWI) [9]. The histological characteristics of AHLE are necrotizing vasculitis of the venules with perivascular hemorrhages and infiltrates with polymorphonuclear cells [75]. Hemorrhagic lesions have been reported in postvaccinal and COVID-19-associated ADEM [66]. Brain MRI investigation, when the clinical context is suggestive, is essential to identify inflammatory lesions and a hemorrhagic component [1,111]. The MRI reveals larger white matter lesions, accompanying edema, and multifocal hemorrhages [112], with a poorer prognosis compared with the classical presentation, despite early intensive treatment [33]. The cerebellar and brainstem involvement, and the presence of gross hemorrhage with mass effect, is correlated with poor prognosis [9]. Reports of patients with AHLE show more elevated levels of inflammatory cytokines and inflammatory markers (CRP, D-dimers, procalcitonin, and serum ferritin) compared with those with classical ADEM [113]. These patients are critically ill, with encephalopathy that deteriorates rapidly to coma and sometimes death. The survivors remain afflicted with significant neurological sequelae [9]. AHLE must be differentiated from posterior reversible encephalopathy syndrome (PRESS), which also presents with parieto-occipital white matter lesions (some of them hemorrhagic) and has also been reported in relation to COVID-19 [35,114].

Lumbar puncture (LP) and CSF can reveal changes in 50–80% of patients—with slightly elevated proteins and lymphocytic pleocytosis, increased pressure, raised levels of myelin basic protein, and, rarely, oligoclonal bands of IgG [32,75,115]. CSF can be normal in up to 60% of cases of ADEM [4,7].

ADEM diagnosis is made based on clinical and radiological features, and the differential diagnosis should include other inflammatory demyelinating diseases of the CNS like multiple sclerosis, neuromyelitis spectrum disorder (NMOSD), autoimmune encephalitis, antiphospholipid antibody syndrome, vasculitis secondary to rheumatic autoimmune disease, tumor, neurosarcoidosis, neuro-Bechet disease, viral encephalopathies, progressive multifocal leukoencephalopathy, adult-onset leukodystrophies, eclampsia, tick-borne etiologies, abscess or viral infections [4,8,14,74,116,117,118,119], and MS. Around 15% of adult patients may experience recurrences, and 25% were reported to have developed multiple sclerosis within 5 years of their initial ADEM event [14,118,119].

The treatment is symptomatic and etiologic, supportive and immunomodulatory, and intended to reduce the morbidity and mortality of ADEM [49]. Intravenous corticosteroids suppress inflammation and aberrant immune responses, and remain the recommended first line of treatment: 1–2 g/day for 3–5 days followed by oral tapering. The response to corticosteroids is favorable in two thirds of cases, and it shortens the duration of the disease and stops further progression. In case of inadequate response, and in refractory cases, therapeutic plasma exchange or IVIg (0.4/kg/body weight) can be recommended according to the protocol applied in other autoimmune neurological diseases [12,117,120,121,122].

Nowadays, in general, the prognosis of ADEM patients is favorable with treatment, with an average recovery period between 1 and 6 months. However, it can result in permanent neurological disability that can burden a patient for the rest of their life [8]. Deaths have been reported in COVID-19-associated ADEM and recovery seems to be incomplete [66]. Sequelae after the initial attack include cognitive impairment, as well as motor and sensory deficits [109]. Mortality is higher among adults compared with the pediatric population [49]. The most important prognostic factors for poor outcomes seem to be the following: the association of COVID-19; ADEM with hemorrhagic features; extensive lesions and brainstem involvement; and the admission to an intensive care unit due to respiratory distress or consciousness impairment [49]. The majority of cases with postvaccinal ADEM have a good evolution with favorable outcomes towards full recovery, as in the case we treated. Only eight reported deaths were found by us in this review (five after infection, three after vaccination). In general, the disease follows a monophasic course, but the evolution towards another demyelinating disease or another multiphasic disease cannot be ruled out; it remains to be seen what the future holds [15].

Despite the increasing number of reported cases of ADEM and AHLE in patients with SARS-CoV-2 infection, this type of neurological complication remains low considering the total number of infections. Numbers of postvaccination cases are even lower. We cannot exclude the possibility that the incidence of adverse postvaccination effects may be slightly higher, as a mild presentation of the disease may remain unreported. In the majority of cases, it is hard to establish if vaccination and the onset of ADEM are coincidental or not, but increasing numbers of vaccinated subjects may support this supposition. A clear link between a certain type of vaccine and increased incidence of ADEM was not observed. Most cases of reported postvaccinal ADEM had a good clinical evolution, even with complete recovery. The absence of encephalopathy seems to be associated with better clinical outcomes in both groups, and its presence is associated with a poor prognosis. The new technology used in the production of vaccines against the SARS-CoV-2 virus, the factors involved in the occurrence of ADEM, the existing gaps in the pathogenic mechanisms, and the lack of clinical studies encourage cautious analyses of cases suspected to be SARS-CoV-2-vaccination-related ADEM. Vaccination is essential for reducing the morbidity and mortality associated with the infection, and additional prospective data are needed for a definite conclusion.

Overall, this study has some limitations: the small sample size with limited published data for some cases (paraclinical investigations, clinical evolution); the retrospective analyses of published reports; and the short follow-up period. In addition, the potential influence of publication bias cannot be excluded, and conclusions may not be representative of the entire population. Another limitation of the study is that not all the patients among those reported with postvaccinal ADEM were tested for SARS-CoV-2. Tests may have become negative after a few weeks and serological tests were not always performed, potentially leading to an underestimation of association between ADEM and COVID-19.

Evidence of immune or immune-mediated CNS damage suggests that neuroinflammation may occur as a long-term consequence of SARS-CoV-2 infection or vaccination, and larger studies with epidemiological and pooled data are needed to check causality.

## 5. Conclusions

The COVID-19 pandemic greatly affects not only the healthcare system, but also the entire socioeconomic system. It is already evident that COVID-19 is a global threat as well as a threat to the CNS, due to its multifactorial pathogenic mechanisms. Attempts to solve this issue have led to the development of different ways to approach the disease, and great effort was made in the development of different vaccines to prevent severe cases of the disease, especially in high-risk categories. Vaccinations are essential to reducing the spread of the COVID-19 pandemic, and the monitoring of adverse events is an important part of the strategic fight against SARS-CoV-2.

In the era of COVID-19, it is mandatory that clinicians should be aware of and remain vigilant to these rare but potential complications following SARS-CoV-2 infection or vaccination, in a suggestive clinical context. A prompt diagnosis and treatment are associated with better prognoses for patients. Although the association between the vaccine and neurologic disease could be coincidental, there is the possibility of a postvaccination neuroinflammatory syndrome given the time sequence of events.

So far, experience suggests that SARS-CoV-2 vaccination is safe. The scarcity of postvaccinal ADEM case reports, and the overall good evolution, should emphasize that the general benefits of vaccination outweigh the risks, and that vaccination programs should continue to be recommended.

## Figures and Tables

**Table 1:** Postvaccine ADEM.

No./Reference	Age /Sex	Vaccine	RT-PCR Test Swab	Neurologic Onset	Neurological Symptoms	Brain Computed Tomography (CT)/MRI Spine MRI	CSF	Other Studies Carried Out	Treatment	Outcome
1. Vogrig, A., et al. [19]	56/F	Pfizer (1st dose)	N	1 week	Horizontal gaze-evoked nystagmus, weakness in left upper limb, left-sided dysmetria, left hemi-ataxic gait	Brain MRI: Hyperintense lesions on FLAIR-weighted images involving left cerebellar peduncle, no contrast enhancement. On a second MRI, new supratentorial areas of hyperintensity on FLAIR sequences were observed.	White blood cells (WBC): normal; Proteins (P): normal; Glucose (G): normal; Anti-SARS-CoV-2 antibodies (−); Oligoclonal bands (OCB) (−); Anti-aquaporin-4 antibodies (AQP4) (−); Anti-myelin oligodendrocyte glycoprotein antibodies (anti-MOG) (−); Microbiological studies (herpes simplex virus (HSV), varicella zoster virus (VZV), human herpes virus 6 (HHV6), Epstein–Barr Virus (EBV), cytomegalovirus (CMV), tick-borne encephalitis (TBE), Borrelia) (−)	Markers of systemic autoimmunity (including antinuclear antibody (ANA), extractable nuclear antigen antibodies (ENA), antineutrophil cytoplasm antibodies (ANCA), antiphospholipid antibodies, complement C3 and C4) (−); Anti-SARS-CoV-2 antibodies in serum (+)	Prednisone 75 mg q.d. with gradual tapering	Favorable: progressive improvement in gait stability
2. Kania, K., et al. [5]	19/F	Moderna	N	2 weeks	Nuchal rigidity, bilateral Babinski signs	Brain MRI: Multiple hyperintense lesions on T2- and FLAIR-weighted images involving both brain hemispheres, pons, medulla oblongata, and cerebellum, some with contrast enhancement. Spine MRI: Hyperintense area from medulla oblongata to Th11 segment and few contrast-enhancing lesions.	WBC: 294/mm^3^ (lymphocytic pleocytosis); Red blood cells (RBC): 77/mm^3^; P: 648 mg/L; Bacterial culture (−); Fungal culture (−); (HSV, VZV, HHV6, EBV, CMV) (−); Neisseria meningitidis (−); Streptococcus pneumoniae (−); Group B streptococcus (−); Hemophilus influenzae (−); Listeria monocytogenes (−)	OCB in serum and CSF (+); AQP4 (−); Anti-MOG (−)	Methylprednisolone	Full recovery
3. Yazdanpanah, F., et al. [20]	37/M	Sinopharm	N	1 month	Bilateral facial nerve paralysis, tetraparesis 2/5 Medical Research Council (MRC)	Brain MRI: Multiple hyperintense lesions on T2- and FLAIR-weighted images involving left cerebral peduncle, pons, and medulla, some with contrast enhancement. Spine MRI: Normal.	WBC: 2/mm^3^; RBC: 32/mm^3^; P: 56 mg/dL; G: 97 mg/dL; OCB (−)	Autoimmune disease markers, vasculitis, viral markers (−)	Methylprednisolone 7 g followed up for 2 weeks for corticosteroid tapering	Full recovery
4. Al-Quliti, K., et al. [21]	56/F	AstraZeneca	N	10 days	Meningism, bilateral-adduction- gaze deficit, tetraparesis 4/5 MRC in upper limbs and 3/5 in lower limbs, diminished deep tendon reflexes	Brain MRI: Multiple bilateral and asymmetric hyperintense lesions on T2- and FLAIR-weighted images involving no contrast enhancement.	WBC: 2/mm^3^; RBC: 32/mm^3^; P: 1.76 g/L; G: 4.62 g/L	-	1 g of methylprednisolone for 5 days	Full recovery
5. Lee, S., et al. [22]	56/M	Pfizer (1st dose)	N	3 days	Confused with behavior changes	Brain MRI: Multiple hyperintense lesions on T2- and FLAIR-weighted images involving bilateral frontal, temporal, and parietal lobes; no contrast enhancement.	WBC: normal; P: normal; G: normal; Microbiological studies (HSV, VZV, HHV, EBV, CMV, etc.) (−); Antibodies against extracellular/intracellular and synaptic neuronal antigens (−); OCB (−)	AQP4 (−); Anti-MOG (−)	1 g of MP for 3 days followed by intravenous immunoglobulins; (IVIG) for 5 days; oral prednisolone in tapering doses over 8 weeks	Favorable: substantial reduction in the signal intensities on follow-up brain MRI
6. Lee, S., et al. [22]	48/M	Pfizer (1st dose)	N	1 day	Left-sided facial pain, numbness in left upper limb	Brain MRI: Hyperintense lesion on T2- and FLAIR-weighted images involving the right and dorsal sides of the pons with contrast enhancement.	WBC: normal; P: normal; G: normal; Bacterial culture (−); Fungal culture (−); OCB (−); IgG index: normal	AQP4 (−); Anti-MOG (−); ANA (−); ENA (−); Angiotensin-converting enzyme (ACE) (−)	1 g of MP for 3 days followed by 1 mg/kg of oral prednisolone	Full recovery
7. Poli, K., et al. [23]	65/M	Pfizer (3rd dose)	Not performed	3 days	Left-sided hemiparesis 4/5 MRC, contralateral dissociated sensory loss, right-sided vestibulocochlear nerve deficit	Brain MRI: Multiple hyperintense lesions on T2- and FLAIR-weighted images involving right cerebellar peduncle, pons, and medulla oblongata with contrast enhancement.	WBC: 54/mm^3^ (lymphocytic pleocytosis); P: normal; G: normal; Screening for bacterial, viral, and fungal neuroinfections (−)	AQP4 (−); Anti-MOG (−); Onconeural antibodies (−); Antiganglioside antibodies (−); Sarcoidosis markers (−); Markers of systemic autoimmunity (−); OCB in serum and CSF (−)	1 g of MP for 5 days followed by 2 g/kg IVIG for 5 days followed by 7 therapeutic plasma exchange (TPE) treatments	Favorable: improvement in neurological status after plasmapheresis
8. Nagaratnam, S.A., et al. [12]	36/F	AstraZeneca (1st dose)	Not performed	2 weeks	Bilateral visual impairment, painful eye movements	Brain MRI: Multiple hyperintense lesions on T2- and FLAIR-weighted images involving subcortical white matter, bilateral internal capsules, pons, and left middle cerebellar peduncle with contrast enhancement. Spine MRI: Normal.	WBC: 59/mm^3^; P: 0.4 g/L; G: 4.8 g/L; OCB (+)	AQP4 (−); Anti-MOG (−)	1 g of MP for 3 days; because the patient’s condition worsened, she received a further course of 3 doses of 1 g of MP followed by 50 mg oral prednisolone in tapering doses	Favorable: significant improvements in visual acuity and color vision
9. Kumar, A., et al. [24]	40/F	AstraZeneca (1st dose)	N	2 weeks	Quadriplegia, reduced sensation to touch and pain in lower limbs, exaggerated deep tendon reflexes, bilateral Babinski sign	Brain MRI: Altered signal on T2- and FLAIR-weighted images involving the visualized cervical cord–medulla region and the right temporal lobe.	WBC: normal; RBC: normal; P: normal; OCB (−); Bacterial culture (−); Fungal culture (−); PCR panels (including VZV, EBV, and CMV) (−); Venereal Disease Research Laboratory (VDRL) (−)	Serum and CSF autoimmune encephalitis panel (−); Anti-MOG (−); ENA (−); Anti-double-stranded deoxyribonucleic acid (DNA) (−)	MP 1 g/day followed by 0.4 g/kg/day IVIG	Patient passed away within a week of admission
10. Miyamoto, K., et al. [25]	54/F	Pfizer (2nd dose)	NA	12 days	Somnolence, urinary retention, bilateral ocular abduction palsy and facial paralysis, sluggish movement, muscle stiffness	Brain MRI: Multiple hyperintense lesions on T2- and FLAIR-weighted images involving cerebral white matter, bilateral basal ganglia, and midbrain.	WBC: 23/uL; P: 31.2 mg/mL; Anti-MOG (+)	Autoimmune encephalitis panel (−); AQP4 (−); Serum anti-MOG (+)	1 g of MP for 3 days followed by 7 plasma exchanges followed by 400 mg/kg/day IVIG for 5 days	Favorable
11. Cao, L., et al. [26]	24/F	Sinopharm	N	2 weeks	Somnolence and memory decline, Mini-Mental State Evaluation (MMSE): 11/30	Brain MRI: Abnormal signals in the bilateral temporal cortex.	WBC: 51 × 10^6^/L; Antibodies to major pathogens (−); Bacterial culture (−); Fungal culture (−); SARS-CoV-2 antibodies (−); AQP4 (−); Anti-MOG (−); Anti-MBP (−); Anti-glial fibrillary acidic protein (GFAP) (−); Autoimmune encephalitis panel (−); Paraneoplastic markers (−)	Serum SARS-CoV-2 antibodies (−); OCB in serum and CSF (−); Human immunodeficiency virus (HIV) (−); Autoimmune vasculitis antibodies (−); Anticardiolipin antibodies (−); Antinuclear antibodies (−)	IVIG 20 g/day for 5 days	Favorable: MMSE 29/30
12. Lazaro, LG., et al. [27]	26/F	Gam-COVID-Vac (1st dose) (human adenovirus viral vector)	NA	4 weeks	Right upper limb weakness, gait ataxia, deferred memory, disorientation, headache, anosognosia, incoherent speech	Brain MRI: Multiple hyperintense lesions on T2- and FLAIR-weighted images.	WBC: 3/uL; P: 50,6 mg/dL; G: 78,3 mg/dL; CSF markers for viral and bacterial agents (−); OCB (+)	Anti-MOG (−); HIV (−); VDRL (−); Hepatitis B/C (−); Brain tissue biopsy (−)	1 g of MP for 5 days	Full recovery
13. Shimizu, M., et al. [28]	88/F	Pfizer (2nd dose)	NA	29 days	Impaired consciousness, gaze-evoked nystagmus	Brain MRI: Multiple hyperintense lesions on T2- and FLAIR-weighted images in the bilateral middle cerebellar peduncles.	CSF markers for viral bacterial and fungal agents (−); OCB (−)	Antinuclear antibodies (−); Autoimmune vasculitis antibodies (−); Onconeural antibodies (−); Antiganglioside antibodies (−)	1 g of MP for 3 days	Favorable
14. Rinaldi, V., et al. [29]	45/M	AstraZeneca (1st dose)	NA	12 days	Nystagmus on lateral gaze bilaterally and right arm pronator drift	Brain MRI: Multiple hyperintense lesions on T2-weighted images in the pons, right cerebellar peduncle, and right thalamus, some with contrast enhancement. Spine MRI: Multiple hyperintense lesions on T2-weighted images at the cervical, dorsal, and conus medullaris levels.	WBC: 44/uL; P: normal; CSF cytology (−); CSF markers for viral and bacterial agents (−); OCB (−);	Herpes viruses (−); HIV (−); Mycoplasma pneumoniae (−); Borrelia burgdorferi (−); AQP4 (−); Anti-MOG (−); Antinuclear antibodies (−); Antineutrophil cytoplasmic antibodies (−); Anticardiolipin antibodies (−)	1 g of methylprednisolone for 5 days followed by oral prednisone tapering	Favorable
15. Maramattom, BV., et al. [30]	46/M	AstraZeneca (1st dose)	N	5 days	Progressive paraparesis	Brain MRI: T2, FLAIR hyperintensities in bilateral middle cerebellar peduncle, pons, medulla, and left thalamocapsular region. Spine MRI: Longitudinally extensive transverse myelitis.	WBC: 63/uL; P: 52 mg/dL; G: 93 mg/dL; CSF markers for viral and bacterial agents (−)	AQP4 (−); Anti-MOG (−); ANCA (−)	IV MP and plasma exchange	Favorable
16. Maramattom, BV., et al. [30]	64/M	ChAdOX1 vaccine (2nd dose)	NA	20 days	Ascending paresthesia in the lower limbs, hand paresthesia, epigastric band-like sensation, leg stiffness; in evolution: spastic paraparesis with paraplegia in the lower limbs	Brain MRI: Bilateral hemispheric hyperintensities of the corticospinal tract. Spine MRI: Multifocal cord hyperintensities	Normal	Lab test for autoimmune encephalitis, paraneoplastic panel: negative	IVIG for 5 days (2 g/kg), IV MP 1 g/day (3 days); after 1 month, rituximab 1 g IV	Favorable
17. Bastide, L., et al. [31]	49/F	ChAdOx1 nCoV-19 AstraZeneca (1st dose)	N	1–2 weeks	Paresthesia in both legs, sphincter dysfunction, hypoesthesia with Th8 level, sensory ataxia	Brain MRI: Multiple hyperintense lesions on FLAIR-weighted images in the periventricular and deep white matter; no contrast enhancement. Spine MRI: Multiple hyperintense lesions on T2-weighted images at the cervical and dorsal levels with contrast enhancement.	WBC: 8/uL; P: 101 mg/dL; CSF markers for viral and bacterial agents (−)	AQP4 (−); Anti-MOG (−); ANA (−); ANCA (−); OCB (−); Serum protein electrophoresis (−); Hepatitis A virus (HAV) (−); Hepatitis B virus (HBV) (−); EBV (−); CMV (−); HIV (−); HSV (−); Syphilis (−); Borrelia b. (−); Toxoplasma (−); John Cunningham (JC) virus (−)	1 g of methylprednisolone for 5 days followed by 5 sessions of therapeutic plasma exchange followed by rituximab 1 gr IV and another course of IV MP	Neurological improvement 8 months later
18. Mousa, H, et al. [32]	44/F	SARS-CoV-2 messenger ribonucleic acid (mRNA) vaccine (1st dose)/possible overlap with M. pneumoniae infection	Negative PCR test	6 days	Blurred vision in the right eye, then in the left eye, numbness and tingling in lower limbs, lower back pain, urinary retention, followed by motor deficit in the lower limbs 1/5, abolished deep tendon reflexes in the lower limbs, diminished sensation at touch and pinprick with a sensory level at T3	T2 spine MRI: A 12 mm lesion at T11-12 level, multifocal and diffuse abnormal signal intensity at C3-C4 and upper thoracic spine suggestive of an active demyelinating plaque. T2 brain MRI: Multiple supratentorial and infratentorial lesions consistent with a demyelinating disease.	WBC: 105 cells, P: 98 mg/dL, myelin basic protein: 10.2 mcg/L, IgG: 11.6 mg/dL, IgA: 1.8 mg/dL, IgM: 1.8 mg/dL, albumin: 59 mg/dL, IgM M. pneumonia Ab: 1943 mg/dL, IgG M. pneumonia Ab: 4.07 mg/dL, EBV DNA qPCR 693 IU/mL, negative oligoclonal bands	Blood lab tests: WBC: 12,100/mL, K: 3.4 mEq/L	5 days of IV pulse therapy, 5 sessions of plasma exchange, and steroid taper	Improvement in visual and urinary symptoms but still with severe neurological deficit with scotoma in both eyes and lower limb deficit
19. Ancău, M, et al. [33]	61/M	ChAdOx1 nCoV-19 vaccine (1st dose)	N	2 days	Headache, apathy, loss of consciousness, generalized seizures, coma	Brain CT diffuse hypodense areas: The right subcortical frontotemporal and right thalamic regions. Brain MRI: Bilateral cortical and subcortical FLAIR hyperintense lesions; hemorrhagic involvement of the basal ganglia.	WBC: 1/µL, negative oligoclonal bands, CSF/serum ratio for albumin of 22.8 × 10^−3^, negative CSF for viral and bacterial agents	AQP4, MOG, ANA, ANCA, antiphospholipid antibodies, neuronal and paraneoplastic antibodies: negative	1 g methylprednisolone IV/day for 5 days followed by 5 sessions of therapeutic plasma exchange with concomitant methylprednisolone administration and subsequent corticosteroid tapering	A slight improvement in alertness, reduction in brain lesions; after 14 weeks of rehabilitation, the patient was still in vegetative state
20. Ancău, M, et al. [33]	25/F	ChAdOx1 nCoV-19 vaccine (1st dose)	N	9 days	Severe cephalgia, thoracic back pain, mild weakness, numbness in lower legs, evolving to paraplegia and anesthesia below T6, abolished deep tendon reflexes, urinary retention	Spine MRI: Longitudinal edema in the thoracic spinal cord with mild contrast enhancement and focal central hemorrhages. Brain MRI: Bihemispheric white matter lesions with contrast enhancement.	Granulocytic pleocytosis: 241 WBC/µL, a highly elevated CSF/serum quotient for albumin of 164.7 × 10^−3^, negative oligoclonal bands	Negative testing for bacterial and viral infections, paraneoplastic antibodies, AQP4, MOG, ANA, ANCA, anti-double-stranded DNA antibodies	1 g methylprednisolone IV/5 days, followed by 7 plasma exchange sessions with concomitant methylprednisolone administration with subsequent corticosteroid tapering	Improvements in cephalgia and in the sensory components but persistent paraplegia at 6-week follow-up
21. Ancău, M, et al. [33]	55/F	ChAdOx1 nCoV-19 vaccine (1st dose)	N	9 days	Dizziness, nausea, meningism followed by severe tetraparesis and coma, increased intracranial pressure, anisocoria, nonreactive mydriasis, hydrocephaly and transtentorial herniation	Brain MRI: Hyperintense and hemorrhagic lesions with frontotemporal distribution but also in the right parietal, temporal, and right occipital lobes, and the left frontobasal region.	Mixed granulocytic and lymphocytic pleocytosis: 10 WBC/µL, negative CSF oligoclonal bands	Negative laboratory testing for infectious agents; negative testing for paraneoplastic antibodies, AQP4, MOG	1 g methylprednisolone IV/5 days with a subsequent tapering of steroids, then a repeated high dose of steroids due to worsening condition	Death
22. Permezel, F., et al. [34]	63/M	Oxford/AstraZeneca COVID-19 vaccine (1st dose)	NA	12 days	Fatigue, vertigo, abdominal pain (ketoacidosis + myocardial infarction) followed by declining cognition, disorientation, impaired attention; later in evolution: poorly responsive, required intubation	Noncontrast brain MRI: Numerous foci in T2 and T2 FLAIR with periventricular and juxtacortical distribution.	NA	Infective causes and malignancy were ruled out	Corticosteroids and plasmapheresis	Death
23. Ahmad, HR., et al. [14]	61/F	Pfizer-BioNTech SARS-CoV-2	N	The symptoms began around the first dose of vaccine	Difficulties in communication due to speech changes, generalized weakness, altered mental status; later in evolution: encephalopathy and tachypnea that required intubation	Brain and cervical spine MRI (without contrast): An acute leukoencephalopathy process affecting the deep white matter extending downward to the brainstem and cerebellum.	P: 61 mg/dL, without other significant changes	K: 3.2 mmol/L, bicarbonate: 11 mmol/L, chloride: 120 mmol/L; cortisol, procalcitonin, glucose level, thyroid function tests, antinuclear antibody, infectious disease panel, paraneoplastic antibodies: normal limits; urine: positive for tetrahydrocannabinol	1 g methylprednisolone IV/5 days in addition to IvIG 2 g/kg in 5 days	The patient regained consciousness, followed commands, and was oriented, but with generalized weakness
24. Stefanou, MI., et al. [35]	47/M	Ad26.COV2.S	N	27 days	Acral paresthesia, flaccid paraparesis with ascending evolution, followed by T6 sensory level, severe tetraparesis	Brain and spine MRI: Neuroimaging findings suggestive of encephalomyelitis (overlapped with GBS).	P: 5.6 g/L, cells: 2/mm^3^	Negative infectious, autoimmune work-up	IVIg 2 gr/kg, IV MP 5 gr	Improvement in the symptoms with mild residual paraparesis at discharge
25. Mumoli, L., et al. [36]	45/M	ChAdOx1 nCoV19	NA; negative IgM and IgG antibodies	7 days	Burning sensations in the back, back pain, followed by numbness and hypoesthesia in the knees, thighs, and perineum, urinary retention, then gait difficulties, febrile status, myalgia, paraparesis, sensory deficit up to D5	First brain CT: Normal. Spinal cord MRI: A central nonexpansive short tau inversion recovery (STIR) signal lesion from D10 to conus without contrast enhancement. Brain MRI: Multiple lesions, hyperintense T2 and FLAIR with bilateral subcortical/cortical gray-white matter lesions without gadolinium enhancement.	Cells: 43 cells, mild hyperproteinorachia, negative oligoclonal bands, negative cultures	Mild leukocytosis, negative extensive serological panel for infections, autoimmune diseases except anti-MOG antibodies 1:2560	IV MP 1 g/day, 5 days	Improvement in sensibility gait symptoms
26. Garg, RK., et al. [37]	56/F	Adenovector-based ChAdOx1 nCoV-19 (COVISHIELDTM) vaccine	NA	2 days	Weakness of the right upper and lower limbs, brisk deep tendon reflexes on the right side, extensive right plantar response	Brain MRI: T2 and FLAIR hyperintensities in the white matter of the left parietal lobe with extension towards corpus callosum with no gadolinium enhancement.	NA	WBC: 21,400/mm^3^ (polymorphs 86%; lymphocyte 12%; eosinophils and monocytes 1% each), C-reactive protein: 3.0 mg/L, without other pathological changes	Oral MP 32 mg/day for 2 weeks, followed by tapered doses (8 mg/week)	Good evolution, independent in daily activities
27. Sivji, M., et al. [38]	49/F	ChAdOx1 nCoV-19 vaccine (AZD1222) (COVISHIELD) (2nd dose)	N	3 weeks	Right lower limb paresthesia with ascending evolution, difficulties in walking and climbing stairs; in evolution: weakness in the right hand, then slurred speech, central facial weakness, sensory impairment below T12 level	Brain MRI: Hyperintense lesions in the right temporal lobe and left posterior lobe.	Normal	Normal blood tests, including autoimmune testing	2 courses of IV MP 1 g/day (5 days) with further tapering of the steroids in the next 10 weeks	Good evolution, without motor deficit at 3-month follow-up
28. El Fargani, R., et al. [39]	34/M	Sinopharm vaccine	N	20 days	Headache, vomiting, photophobia, acute febrile confusion; Glasgow Coma Scale (GCS): 13 points	Brain CT: Left temporal hypodensity. Brain MRI: Signal defect in the supratentorial and infratentorial white matter, basal ganglia lesions.	37 elements/mm^3^, predominantly lymphocytes; negative test for infectious diseases, sterile culture, normal levels of proteins and glucose	WBC: 17,820/uL with neutrophilia (13,250/uL)	IV MP 1 g/day (5 days), then oral MP 1 g/kg/day but without improvement, followed by 5 sessions of therapeutic plasma exchange	Clinical improvement

**Table 2:** Postinfectious ADEM.

Study	Age /Sex	RT-PCR Test Swab	COVID-19 Severity	Neurological Onset	Neurological Symptoms	Brain CT/MRI Spine MRI	CSF	Other Studies Carried Out	Treatment	Outcome
1. Parsons, T., et al. [40]	51/F	P	Severe—required intubation	NA	Unresponsive (GCS 3)	Brain MRI (day 24): Hyperintense lesions on FLAIR imaging in deep hemispheric and juxtacortical white matter with mild contrast enhancement (repeated during hospitalization, showing an increase in the number and distribution of FLAIR lesions).	WBC: 1/mm^3^; RBC: 2095/mm^3^; P: 62 mg/dL; G: 56 mg/dL; Bacterial culture (−); Fungal culture (−); PCR panel (including HSV, VZV, EBV, and CMV) (−); PCR for SARS-CoV-2 (−)	OCB in serum and CSF (+); ANA (−); ANCA (−); AQP4 (−); HIV (−); Syphilis (−)	MP 1 g/day, 5 days; IVIG 0.4 g/kg/day, 5 days from day 31	Favorable: alertness improved gradually, followed simple commands on day 36; able to speak on day 39; fully oriented on day 59
2. Novi, G., et al. [41]	64/F	N	Influenza-like syndrome	3–4 weeks after COVID-19 symptom onset	Irritability, headache, severe vision loss, right abdominal sensory level, left lower limb hyperreflexia, Babinski sign on left side	Brain MRI: Multiple T1 post-gadolinium-enhancing lesions; bilateral optic nerve enhancement. Spine MRI: A spinal cord lesion at the T8 level.	WBC: 22/mm^3^ (lymphocytic pleocytosis); P: 452 mg/L; PCR for SARS-CoV-2 (+)	OCB in serum and CSF (+); AQP4 (−); Anti-MOG (−)	MP 1 g/day, 5 days, tapered with oral prednisone 75 mg/d associated with IVIG (2 g/kg in 5 days)	Favorable: progressive recovery of visual acuity
3. Neppala, S., et al. [42]	68/M	P	Severe—required intubation	NA	Unresponsive (GCS 3)	Brain MRI: Bilateral multifocal white matter FLAIR signal hyperintensities; no contrast enhancement. Spine MRI: No abnormalities in the spinal cord.	WBC: 3/mm^3^; RBC: 50/mm^3^; P: 28 mg/dL; G: 109 mg/dL; CSF cultures (−); PCR panels (including COVID-19 RNA, HSV, VZV, EBV, and CMV) (−)	OCB in serum and CSF (−); Anti-MOG (−); ANA (−); ANCA (−); HIV (−); Syphilis (−); AFB (−)	40 mg IV methylprednisolone for a few weeks	Favorable: complete resolution of motor aphasia, and muscle strength improved to 4/5 MRC
4. Neppala, S., et al. [42]	49/M	P	Severe—required intubation	NA	Unresponsive (GCS 3)	Brain MRI: Bilateral multifocal white matter FLAIR signal hyperintensities; no contrast enhancement. Spine MRI: No abnormalities in the spinal cord.	WBC: 9/mm^3^; RBC: 1100/mm^3^; P: 91 mg/dL; G: 66 mg/dL; CSF cultures (−); PCR panels (COVID-19 RNA, HSV, VZV, EBV, and CMV) (−)	OCB in serum and CSF (−); Anti-MOG (−); ANA (−); ANCA (−); HIV (−); Syphilis (−)	40 mg IV methylprednisolone for a few weeks	Favorable: complete resolution of motor aphasia, and muscle strength improved to 3/5 MRC
5. Zelada-Riíos, L. et al. [13]	35/M	NA	Mild: dry cough and fatigue resolved in 3 days	7 days after COVID-19 symptom onset	Nystagmus, bilateral VI cranial nerve palsy, absent gag reflex, dysarthria, tetraparesis 4/5 MRC, hyperreflexia in lower limbs, ataxia, gait impairment	Brain MRI: Multiple disseminated T2 and FLAIR hyperintensities; minimal contrast enhancement. Spine MRI: Diffuse, confluent intramedullary lesions with faint contrast enhancement between C5 and C7.	WBC: normal; RBC: normal; P: 47 mg/dL; G: normal	Serum anti-SARS-CoV-2 IgM/IgG antibodies (+); OCB in serum and CSF (−); Anti-MOG (−)	Two cycles of MP 1 g/day for 5 days tapered with oral prednisone	Favorable: improvement in neurological symptoms after 10 days
6. Berrichi, S., et al. [43]	38/F	P	Cough, fever, oxygen saturation of 88% on ambient air	2 weeks after COVID-19 symptom onset	Incoherent speech, aggressiveness, visual and auditory hallucination, flaccid paraparesis, umbilicus sensory level, urinary retention	Brain MRI: FLAIR nodular hyperintensities in the juxtacortical frontal and temporal white matter, left thalamus, and brainstem. Spine MRI: T2 hyperintensities with contrast enhancement along the posterior column of the cervical spinal cord.	WBC: 17/mm^3^; IgG index 1.2 OCB (+)	-	An intravenous injection of 400 mg of Tocilizumab, and high doses of methylprednisolone	Favorable: neurological symptoms slowly regressed
7. Ghosh, R., et al. [44]	34/F	P	Severe: fever, anorexia, weakness, headache	NA	Myoclonus, gait ataxia (neurological status worsened over the next 3 days; she became unresponsive to external stimuli)	Brain MRI: Multiple disseminated T2 and FLAIR hyperintensities in both fronto-parieto-occipital subcortical regions, bilateral thalami, red nuclei, and basis points of brainstem. Spine MRI: Normal.	WBC: 9/mm^3^ (all lymphocytes); P: 89 mg/dL; CSF was tested for viral, bacterial, and parasitic infections, tuberculosis, paraneoplastic, autoimmune encephalitis (−)	AQP4 (−), anti-MOG (−); paired sera for viral, bacterial, and parasitic infections, tuberculosis, paraneoplastic, autoimmune encephalitis (−)	MP 1 g/d for 5 days and IVIG (2 g/kg in 5 days) on 22nd day of admission	Favorable: movement disorders persisted after MP; myoclonus and gait ataxia improved significantly after IVIG
8. Langley, L., et al. [45]	53/M	P	Severe—required intubation	NA	No verbal response to pain, motor response was limited to right-hand twitching, no limb reflexes	Brain MRI: Multiple hyperintense lesions within the subcortical and deep white matter of the frontoparietal lobes bilaterally; no contrast enhancement.	C: normal;P: normal CSF cultures (−); PCR panel (including HSV, VZV, EBV, adenovirus, and CMV) (−)	OCB in serum and CSF (+)	MP 1 g/d for 3 days and tapered with oral prednisolone	Favorable: at discharge, muscle strength had improved to 3/5 MRC in left arm and 5/5 MRC in lower limbs
9. McCuddy, M., et al. [46]	37/F	P	Severe—required intubation	Diagnosis of ADEM on day 22 of hospitalization	Tetraparesis with paraplegia	Brain MRI: Multiple T2 hyperintense lesions involving cerebral white matter, corpus callosum, pons, and medulla; some lesions present contrast enhancement.	WBC: 2/mm^3^; P: 95 mg/dL; G: 85 mg/dL; Meningitis/encephalitis panel (−); OCB (−); PCR for SARS-CoV-2 (−)	NA	Decadron 20 mg iv 5 days and 10 mg 5 days	Partially favorable: 50 days after admission, presented with partial return of strength in upper extremities and regaining of some function in distal lower limbs
10. McCuddy, M., et al. [46]	56/M	P	Severe—required intubation	Diagnosis of ADEM on day 20 of hospitalization	Unresponsive	Brain MRI: Multiple T2 hyperintense lesions involving cerebral white matter and deep bilateral cerebellum.	WBC: 1/mm^3^; P: 55 mg/dL; G: 112 mg/dL; CSF culture (−); Lyme disease (−); Multiple sclerosis (MS) panel (−); VDRL (−); OCB (−); PCR for SARS-CoV-2 (−)	NA	Solumedrol 1 g/day, 5 days and IVIG 25 g/day, 3 days	Unfavorable: not opening eyes, unresponsive to painful stimuli, remains on ventilation
11. McCuddy, M., et al. [46]	70/F	P	Severe—required intubation	Diagnosis of ADEM on day 16 of hospitalization	Unresponsive to verbal stimuli, withdraws to pain	Brain MRI: Multiple T2 hyperintense lesions involving cerebral white matter, corpus callosum, and pons with minimum enhancement.	WBC: 0/mm^3^; P: 63 mg/dL; G: 87 mg/dL; Meningitis/encephalitis panel (−); CSF culture (−); Lyme disease (−); MS panel (−); VDRL (−); OCB (−); PCR for SARS-CoV-2 (−)	NA	Solumedrol 1 g/day, 5 days and IVIG 25 g/day, 3 days	Partially favorable: opened eyes spontaneously, flexion withdrawal, weaning from the ventilator
12. Lopes, C.C.B., et al. [47]	59/F	P	Severe—required intubation	NA	Patient in coma, with asymmetric flexor motor responses, hyperreflexia, and bilateral Babinski sign	Brain MRI: Hyperintensity on T2- and FLAIR-weighted images in the cerebral and cerebellar white matter and corpus callosum.	WBC: normal; RBC: normal; P: normal; G: normal; CSF culture (−); PCR for SARS-CoV-2 (−); OCB (−)	H1N1 (−)	-	Patient died of systemic complication
13. Lopes, C.C.B., et al. [47]	41/M	P	Severe—required intubation	20 days after COVID-19 symptom onset	Poor verbal interaction, decreased spontaneous movement of the four limbs, with normal withdrawal response to pain	Brain MRI: Hyperintense lesions on FLAIR-weighted images in centrum semiovale, bilaterally, right thalamus, globus pallidus bilateral, and bilateral internal capsule.	WBC: normal; RBC: normal; P: normal; G: normal; Negative microbiologic analysis; OCB (−); PCR for SARS-CoV-2 (−)	-	NA	Favorable: after two weeks, patient had mild attentional and executive dysfunction
14. Kumar, A., et al. [48]	35/F	P N at admission	Anosmia and ageusia	2 months	Gait instability, later became unarousable to noxious stimulation	Brain MRI: Symmetric hyperintense lesions on FLAIR-weighted images involving bilateral cerebral peduncles. On the second MRI, lesions had progressed. Spine MRI: Normal.	WBC: 1/mm^3^; RBC: 0/mm^3^; P: 22 mg/dL; G: 76 mg/dL; Meningitis/encephalitis panel (−); CSF culture (−); OCB (−)	Myelin basic protein (MBP) (+); AQP4 (−); Anti-MOG (−); ANA (−); Antimitochondrial antibodies (AMA) (−); Anti-JO-1 (−); Anti-liver kidney microsomal antibody (LKM) (−); Antiphospholipid antibodies and IgG (−); Ceruloplasmin (−); Lyme serologies (−)	MP 1 mg/kg for 5 days and IVIG (2 g/kg in 3 days); as the neurological status did not improve, she received 5 days of PLEX	Unfavorable: after 48 days of hospitalization, her condition did not improve and she was transferred to a long-term care facility
15. Oumerzouk, J., et al. [49]	58/M	P	Mild: nausea and vomiting	3 days after COVID-19 symptom onset	Left central vestibular syndrome and left lateropulsion	Brain MRI: Hyperintense lesions on FLAIR-weighted images in right thalamus, left cerebellar, and right parietal regions.	WBC: normal; RBC: normal; P: normal; G: normal; CSF culture (−); OCB (−); PCR for SARS-CoV-2 (−)	EMG: normal	MP 1 g/d for 5 days followed by oral prednisone 1 mg/kg/day, which was then gradually tapered over 10 weeks	Favorable with rapid regression of the symptoms; a brain MRI performed 15 days after full recovery was completely normal
16. Oumerzouk, J., et al. [49]	25/M	P	Moderate/severe: fever, cough, respiratory distress	2 weeks after COVID-19 symptom onset	Rhythmic movements of the right arm, vertigo, Romberg’s sign positive followed by a rapid deterioration in neurological status (GCS 5)	Brain MRI: Hyperintense lesions on T2- and FLAIR-weighted images in left temporal and bilateral frontoparietal lobes; multiple punctiform signal voids in T2 of the two cerebral hemispheres and vermis.	WBC: normal; RBC: normal; P: normal; G: normal	Hepatitis B and C (−); Toxoplasmosis (−); HIV (−); Syphilis (−); stereotaxic brain biopsy: demyelination and perivenular inflammation without signs of a neoplasm	Intravenous methylprednisolone 1 g	Patient died after 7 days of hospitalization
17. Oumerzouk, J., et al. [49]	54/F	P	Moderate/severe: fever, cough, headache, dyspnea	9 days after COVID-19 symptom onset	Altered level of consciousness, cerebellar syndrome, numbness of the four limbs, spastic tetraparesis	Brain MRI: Multiple confluent hyperintense lesions on T2- and FLAIR-weighted images involving cerebral white matter, left middle cerebellar peduncles, thalamus, and lenticular nucleus.	WBC: 4/mm^3^; RBC: 0/mm^3^; P: 36 mg/dL; G: 59 mg/dL; CSF culture (−); OCB (−)	-	MP 1 g/d for 3 days followed by oral prednisone 1 mg/kg/day, for 6 weeks, which was then gradually tapered	Favorable: after 3 months, patient presented left spastic hemiparesis with a left cerebellar syndrome
18. Abdi, S., et al. [50]	58/M	P	No complaints of pulmonary symptoms	NA	Altered level of consciousness, impaired movement of the left upper limb	Brain MRI: Multiple confluent hyperintense lesions on FLAIR-weighted images involving cerebral white matter, deep gray matter, and midbrain, without prominent enhancement on T1-weighted images.	WBC: 0/mm^3^; P: 15 mg/dL; G: 105 mg/dL; PCR panels (including HSV, VZV, EBV, and CMV) (−); OCB (−)	Tuberculosis (−); Brucella antibodies (−); HIV (−)	Intravenous dexamethasone 8 mg/day	Favorable: improvement in mental status after two days
19. Esmaeili, S., et al. [51]	67/M	P	Severe—required intubation	2 days after COVID-19 symptom onset	Altered level of consciousness, could not speak consistently, and could not obey simple tasks	Brain MRI: Hyperintense lesions on FLAIR-weighted images involving cerebral white matter, middle cerebellar peduncles, corpus callosum, the basal ganglia, thalami, midbrain, and pons with enhancements in the midbrain and left parietal lobe.	WBC: normal; RBC: normal; P: normal; G: normal; PCR panels (including HSV, VZV, EBV, and CMV) (−); PCR for SARS-CoV-2 (−); OCB (−)	-	MP 1 g/day for 5 days followed by IVIG (0.4 g/kg in 5 days)	The patient died after 4 weeks of hospitalization
20. Rossi, T., et al. [52]	59/F	N	Flu-like syndrome	4 weeks after COVID-19 symptom onset	Severe visual field defect in both eyes, color vision deficiency, pain in all directions of gaze, left lower limb hyperreflexia with Babinski sign, and right-sided sensory impairment	Brain MRI: Bilateral optic nerve enhancement. Spine MRI: D7-D8 spinal cord lesion with enhancements.	WBC: 22/mm^3^ (lymphocytic pleocytosis); P: 452 mg/L; G: normal; PCR for SARS-CoV-2 (+)	-	MP 1 g/day for 5 days followed by IVIG (2 g/kg in 5 days) and oral prednisone 75 mg/day (that was subsequently tapered)	Favorable: ten days later, vision improved and the number of gadolinium-enhancing lesions reduced in MRI
21. Gelibter, S., et al. [53]	53/M	P	Mild respiratory symptoms and fever	2 weeks after COVID-19 symptom onset	Bilateral blindness, altered level of consciousness, dysarthria, ophthalmoplegia, left hemiparesis, ataxia, left upper limb dystonia	Brain MRI: Hyperintense lesions on FLAIR-weighted images involving bilateral cerebral white matter, with incomplete gadolinium enhancement. Spine MRI: Dorsal enhancing lesions.	WBC: 1/mm^3^; P: 74 mg/dL; G: normal; PCR panels (including HSV, VZV, EBV, and CMV) (−); PCR for SARS-CoV-2 (−); AQP4 (−); Anti-MOG (−); OCB (−)	Anti-Hu, anti-Yo, anti-Ri, anti-Tr, anti-CV2, anti-Ma proteins, anti-amphiphysin, and anti-glutamic acid decarboxylase (GAD) tested negative	MP 1 g/day for 7 days, followed by intravenous tapering for a total of 10.5 g and IVIG (2 g/kg in 5 days); 2 more sessions of IVIG were performed	Mild recovery
22. Verriello, L., et al. [54]	58/M	N	Mild respiratory symptoms	4 weeks after COVID-19 symptom onset	Ataxic gait, left-sided dysmetria, mild left hemiparesis	Brain MRI: Hyperintense lesions on T2- and FLAIR-weighted images involving deep and periventricular white matter of frontoparietal and occipital lobes, corpus callosum, cerebellum, and brainstem.	WBC: normal; P: 508 mg/L; OCB (−); CSF markers for viral and bacterial agents (−); PCR for SARS-CoV-2 (−)	Serum anti-SARS-CoV-2 IgG antibodies (+)	MP 1 g/day for 5 days tapered with oral prednisone	Mild recovery
23. Shahmirzaei, S., et al. [55]	30/M	N	No symptoms	Not clear	Gait ataxia, right-sided internuclear ophthalmoparesis, confusion	Brain MRI: Hyperintense lesions on T2- and FLAIR-weighted images suggestive of acute disseminated encephalomyelitis.	WBC: 0/mm^3^; RBC: 16/mm^3^; P: 45.7 mg/dL; G: 58 mg/dL; CSF culture (−); OCB (+)	AQP4 (−); Anti-MOG (−); IgG anti-SARS-CoV-2 antibodies in serum (+)	MP 1 g/day for 5 days followed by rituximab 1 g iv	Favorable
24. Zoghi, A., et al. [56]	21/M	N	Fever, nonproductive cough, and a sore throat	2 weeks	Tetraparesis 4+/5 MRC in upper limbs and 2/5 in lower limbs, urinary retention, T8 sensory level	Brain MRI: Long corticospinal tract lesions in internal capsules extending to the cerebral peduncles and pons and corpus callosum hyperintensity signal abnormalities on T2- and FLAIR-weighted images. Cervical spine MRI: Longitudinally extensive transverse myelitis.	WBC: 150/mm^3^ (lymphocytic pleocytosis); P: 281 mg/dL; G: 34 mg/dL; Bacterial culture (−); Fungal culture (−); PCR panels (including HSV, HI, etc.); AQP4 (−); Anti-MOG (−); PCR for SARS-CoV-2 (−)	HIV (−); Hepatitis B and C (−); antiphospholipid antibodies (−); ACE (−); human leukocyte antigen (HLA) B5 and B51 (−); Serum AQP4 (−); Serum anti-MOG (−)	Daily plasma exchange for 5 days	Slow recovery
25. Zanin, L., et al. [57]	54/F	P	Anosmia and ageusia a few days prior, severe hypoxia after admission, required intubation	NA	GCS of 12 (E3 M6 V3), without focal sensorimotor deficits	Brain and spine MRI: Hyperintense lesions on T2-weighted images involving periventricular white matter, bulbo–medullary junction, cervical and dorsal spinal cord.	WBC: normal; P: normal; G: normal; PCR panels (including SARS-CoV-2, HSV, VVZ, CMV)	-	Dexamethasone 20 mg/day for 10 days followed by 10 mg/day for 10 days	Favorable: the patient was transferred to rehabilitation, without sensorimotor deficits after 12 days
26. Utukuri, P.S., et al. [58]	44/M	P	None	NA	Urinary retention, paraparesis, dysarthria, bilateral arm ataxia	Brain MRI: Nonenhancing, hyperintense lesions on T2-weighted images involving periventricular and juxtacortical white matter. Spine MRI: Nonenhancing, hyperintense lesions on T2-weighted images throughout cervical and thoracic spinal cord.	WBC: 6/mm^3^; P: 36 mg/dL; Bacterial culture (−); Viral PCR studies (−); PCR for SARS-CoV-2 (+); OCB (−)	-	IVMP followed by IVIG	Modest improvement
27. Karsidag, S., et al. [59]	18/F	P	Fever, diarrhea, fatigue, and hyposmia	2 weeks after COVID-19 symptom onset	Horizontal nystagmus, truncal ataxia, and cerebellar dysmetria on both sides	Brain MRI: Hyperintense lesions on T2- and FLAIR-weighted images involving bilateral periventricular and subcortical white matter, some with contrast enhancement.	WBC: 0/mm^3^; P: 11 mg/dL; G: 52 mg/dL; Bacterial culture (−); Fungal culture (−); Viral PCR studies (including SARS-CoV-2) (−); OCB (−)	-	MP 1 g/day for 7 days	Started to walk independently
28. Karsidag, S., et al. [59]	42/F	Diagnosis of COVID-19 one month prior		3 weeks after COVID-19 symptom onset	Paresthesia in the left mandibular branch of the trigeminal nerve, left hemiparesis 4/5 MRC, Babinski sign was found positive on the left foot	Brain MRI: Hyperintense lesions on T2- and FLAIR-weighted images involving bilateral periventricular white matter, some with contrast enhancement.	WBC: 0/mm^3^; P: 22 mg/dL; G: 48 mg/dL; Bacterial culture (−); Fungal culture (−); Viral PCR studies (including SARS-CoV-2) (−); OCB (−); Mycobacterium tuberculosis (−); Syphilis (−); AQP4 (−)	-	MP 1 g/day for 7 days	Favorable: symptoms improved
29. Karsidag, S., et al. [59]	32/F	Diagnosis of COVID-19 four months prior		4 months after COVID-19 symptom onset	Hypoesthesia in the mandibular branch of the trigeminal nerve and weakness in the right leg and Babinski sign on the right side after two months	Brain MRI: Hyperintense lesions on T2- and FLAIR-weighted images involving bilateral periventricular white matter, cerebellum, and left pontocerebellar junction, some with contrast enhancement. Spine MRI after 2 months: New abnormal signal intensities with contrast enhancement at the C 6–7 levels.	WBC: 0/mm^3^; P: 30 mg/dL; G: 80 mg/dL; Bacterial culture (−); Fungal culture (−); Viral PCR studies (−); PCR for SARS-CoV-2 (+); OCB (+); Mycobacterium tuberculosis (−); Syphilis (−); AQP4 (−); ANA (−); ENA (−)	-	64 mg of oral methylprednisolone; after 2 months, she received MP 1 g/day for 10 days	Full recovery
30. El Beltagi, A.H., et al. [60]	25/M	P	Flu-like symptoms	3 weeks after COVID-19 symptom onset	Quadriparesis 4/5 MRC, ataxia	Brain MRI: Hyperintense lesions on T2- and FLAIR-weighted images involving both brain hemispheres and midbrain, some with contrast enhancement. Spine MRI: T2 hyperintensities along the cervicodorsal spinal cord.	Lymphocytic pleocytosis with elevated glucose and protein levels; Bacterial culture (−); Viral PCR studies (−); Mycobacterium tuberculosis (−); Autoimmune panel (−)	-	NA	NA
31. Freire- Álvarez, E., et al. [61]	39/M	P	Fatigue, fever, headache	2 weeks after COVID-19 symptom onset	Minimal stiff neck, drowsiness, paraphasia	Brain MRI: Hyperintensities in the cortical and subcortical right frontal regions, right thalamus and mammillary body, bilateral temporal lobes, and cerebral peduncles.	WBC: 20/mm^3^; P: 198 mg/dL; G: 48 mg/dL; Bacterial culture (−); Fungal culture (−); Viral PCR studies including SARS-CoV-2 (−)	HIV (−); Treponema pallidum (−); Borrelia burgdorferi (−)	0.4 g/kg/day IVIG for 5 days, iv. tocilizumab for 3 days	Without significant neurological improvement
32. Umapathia, T., et al. [62]	59/M	P	Asymptomatic	Unknown	Encephalopathy, quadriplegia	Brain MRI: Hyperintense lesions on T2- and FLAIR-weighted images involving periventricular and deep white matter bilaterally, cerebral peduncles, pons, middle cerebellar peduncles, and cerebellar white matter.	WBC: 6/mm^3^; RBC: 22/mm^3^; P: 0.56 g/L; G: 2.7 mmol/; Bacterial culture (−); Fungal culture (−); Viral PCR studies including SARS-CoV-2 (−); OCB (−); Autoimmune encephalitis panel (−)	Syphilis (−); Autoimmune encephalitis panel (−); AQP4 (−); Anti-MOG (−); ANA (−); Paraneoplastic panel (−)	MP 1 g/day for 7 days	Partially favorable: at 3.5 months of illness, he was able to open eyes spontaneously
33. Kızılırmak, R., et al. [63]	32/F	P	Nausea	2 weeks after COVID-19 symptom onset	Altered level of consciousness, GCS of 9, neck stiffness, right upper extremity spasticity	Brain MRI: Hyperintense lesions on T2- and FLAIR-weighted images involving left frontal subcortical white matter and corpus callosum genus.	WBC: 10/mm^3^; P: 55 mg/dL; Bacterial culture (−); Viral PCR studies including SARS-CoV-2 (−); OCB (−)	-	MP 1 g/day for 5 days and 6 cycles of plasmapheresis every other day	Partial improvement in the symptoms
34. Benevides, M.L., et al. [64]	50/F	P	Myalgia, fever, vomiting, headache	3 days after COVID-19 symptom onset	2 episodes of grand mal seizures	Brain MRI: Hypointense lesions on the right temporal lobe, left parietal lobe, and both occipital lobes in SWI.	WBC: 8/mm^3^; RBC: 768/mm^3^; P: 150 mg/dL; G: 67 mg/dL; Bacterial culture (−); Fungal culture (−); PCR panels (including SARS-CoV-2, HSV, VVZ, CMV)	HIV (−); Hepatitis B and C (−); Syphilis (−); antinuclear antibodies (−)	MP 1 g/day for 5 days	Full recovery
35. Varadan, B., et al. [9]	46/M	P prior admission	Fever, dyspnea	5 weeks after COVID-19 symptom onset	Altered mental status, left hemiparesis 0/5 MRC in upper limb and 3/5 MRC in lower limb	Brain MRI: Hyperintense lesions on T2- and FLAIR-weighted images involving bilateral frontal, parietal lobes, left thalamus, left cerebral peduncle, and medulla. Second MRI: Progression in number and size of the lesions with florid intralesional hemorrhage.	Lymphocytic pleocytosis with increased protein; Bacterial culture (−); Fungal culture (−)	-	MP 1 g/day for 5 days	Patient died
36. Chalil, A., et al. [65]	48/F	P	Myalgia, dry cough, dyspnea, fever	2 weeks after COVID-19 symptom onset	Equal and nonreactive pupils, absent gag and corneal reflexes	Brain CT: Bilateral parietal and occipital intraparenchymal hemorrhage with intraventricular extension. Brain MRI: Cortical gadolinium enhancement with hyperintense T2 and FLAIR signal surrounding the hemorrhage.	WBC: 76/mm^3^; PCR for SARS-CoV-2 (−)	-	Briefly treated with tocilizumab	Patient is undergoing rehabilitation with severe neurologic deficits
37. Paterson, R. W., et al. [66]	52/M	P	Myalgia, cough, dyspnea, fever	Approx. 2 weeks after COVID-19 symptom onset	Impaired consciousness (responding to pain only)	Brain MRI: Bilateral white matter changes with hemorrhage.	OCB (−); Viral PCR panel (−)	-	Supportive treatment	Slow recovery
38. Dixon, L., et al. [67]	59/F	P	Fever, cough, and headache	10 days	Impaired consciousness, at admission GCS 11 of 15, and after 12 h the patient’s GCS fell to 5 (E1,V1,M3)	Brain MRI: Extensive abnormal signal and microhemorrhage within the dorsolateral putamina, ventrolateral thalamic nuclei, subinsular regions, corpus callosum, cingulate gyri, and subcortical white matter with peripheral contrast enhancement.	WBC: 4/mm^3^; P: 2.3 g/L; Bacterial culture (−); Viral PCR panel (including SARS-CoV-2, HSV, VVZ, CMV, etc.)	-	High dose of dexamethasone	The patient died after 10 days of hospitalization
39. Paterson, R. W., et al. [66]	52/M	P	Cough, dyspnea, fever	NA	Headache, flaccid tetraparesis, areflexia; after 3 days, developed dysphagia and ophthalmoplegia	Brain MRI: Multifocal confluent lesions in internal and external capsules, splenium, and deep white matter which increased in size and showed multiple microhemorrhages after 5 days. Spine MRI: Gadolinium enhancement of the cervical and lumbar roots.	WBC: 0/mm^3^; P: increased level; PCR for SARS-CoV-2 (−)	Nerve conduction studies: supported a diagnosis of GBS	MP 1 g/day for 5 days	Neurological improvement following treatment on day 3
40. Paterson, R. W., et al. [66]	47/F	P	Cough, fever, and shortness of breath	1 week after COVID-19 symptom onset	Severe headache, left-sided numbness, left-sided weakness	Brain MRI: Right hemispheric edema with a leading edge on contrast imaging; small areas of T2 hyperintense changes in the left hemisphere.	-	Brain biopsy showed perivenular inflammation supporting aggressive hyperacute ADEM	MP 1 g/day for 5 days, followed by oral prednisolone 60 mg daily and 5 days of IVIG, hemicraniectomy	Slow recovery
41. Haqiqi, A., et al. [68]	56/M	P	Flu-like symptoms	2–3 weeks after COVID-19 symptom onset	Impaired consciousness	Brain MRI on day 27: Normal. Brain MRI on day 60: Extensive abnormal signal throughout the white matter bilaterally with hemorrhage compatible with hemorrhagic leukoencephalitis.	WBC: 8/mm^3^; RBC: 6/mm^3^; P: 0.71 g/L; G: 4.3 mmol/L; Bacterial culture (−); PCR panels (including SARS-CoV-2, HSV, VVZ, CMV, etc.)	West Nile virus serum IgM (−); OCB in serum and CSF (+); CMV (−); Hepatitis A (−); Hepatitis B (−); Hepatitis C (−); HIV (−); EBV (−)	NA	Patient was discharged to a neurorehabilitation center
42. Fitouchi, S et al. [69]	54/M	P	ARDS, respiratory support, thoracic CT scan: lesions up to 50% of the lung parenchyma	10 days after COVID-19 onset	GCS 5	Brain MRI: Nodular FLAIR hyperintense lesions, right optic nerve, subcortical white matter, bilateral corticospinal tracts, located in parietal and occipital lobes with mild contrast enhancement; mild mass effect; normal medullar MRI.	Lumbar puncture (LP): normal cell count, normal protein, glucose, and lactate dehydrogenase (LDH) levels; negative bacterial cultures, SARS-CoV-2 (−), HSV (−), VZV (−), CMV (−), EBV (−), HHV6 (−), enterovirus (−), HIV (−), OCB in serum and CSF (+)	Anti-N-Methyl-D-Aspartate (NMDA), α-amino-3-hydroxy-5-methyl-4-isoxazolepropionic acid (AMPA), aminobutyric acid (GABA), dipeptidyl-peptidase–like protein 6 (DPPX), leucine-rich glioma inactivated 1 (Lgi1), contactin-associated protein like 2 (Caspr2), glycine, and GFAP (−)	MP 1 g/day from day 15—no response TPE from day 21 (5 sessions)—no response Rituximab 1 g (days 28 and 42)—no response	Discharged with persistent vegetative state
43. Handa, R., et al. [70]	33/M	P	Fever	2 days after COVID-19 symptom onset	Generalized tonic–clonic seizures, GCS of 7 (E2 V1 M4), tetraparesis, areflexia	Brain MRI: Symmetrical FLAIR hyperintensities involving bilateral subcortical frontoparietal lobes, corpus callosum, medulla, and visualized cervical cord with petechial hemorrhages.	WBC: 5/mm^3^; P: 35 mg/dL; G: 75 mg/dL; Bacterial culture (−); HSV 1 and 2 (−); Tuberculosis (−); Cryptococcal antigen (−)	-	MP 1 g/day for 5 days	Favorable: patient became conscious and responsive
44. Yong, MH, et al. [71]	61/M	P	Fever, tachypnea; in evolution: respiratory failure	18 days after COVID-19 onset	GCS 3	Brain MRI: Asymmetrical, multifocal lesions in the subcortical white matter, overlying cortex; bilateral thalamus and cerebellar hemisphere involvement; mass effect of largest lesion; innumerable widespread petechial hemorrhages; incomplete ring-like enhancement of the thalamic lesions.	NA (increased intracranial pressure)	Lymphopenia (0.58 × 10^9^/L); LDH 2239 u/L, Ferritin 6575 μg/L, C-Reactive-Proteine (CRP) 228 mg/L, D-dimer > 32 mg/L, and interleukin-6 level 154 ng/mL	TPE (1 session followed by citrate toxicity) IVIg 2 g/kg in 5 days MP 1 g/day (5 days)	Improved to GCS 13 Tetraparetic, dysphasic
45. Baghal, M., et al. [72]	56/M	Recently discharged after being treated for SARS-CoV-2 infection		1–2 weeks after COVID-19 symptom onset	Right-sided unilateral loss of vision, severe slurring of speech, loss of ability to walk without assistance	Brain MRI: Signs consistent with acute hemorrhagic necrotizing encephalitis.	WBC: 10/mm^3^; P: 300 mg/L; G: 5.2 mmol/L; Bacterial culture (−)	Blood culture (−)	1 g IVIG for 3 days followed by oral prednisone 60 mg/day with gradual tapering over 6 weeks	Favorable: gradual improvements in weakness and visual impairment

**Table 3:** All the characteristics of the postinfection and postvaccination ADEM patients.

Variables	Postinfection *n* = 45	Postvaccination *n* = 29	*p*-Value
Male/Female Sex no. (%)	26 (57.78%) 19 (42.22%)	13 (44.82%) 16 (55.18%)	0.27
COVID-19 Infection Severity, no. (%)			
Asymptomatic	4 (8.89%)	-	0.21
Mild	16 (35.56%)	-	*0.01*
Moderate	11 (24.44%)	-	*0.04*
Severe	14 (31.11%)	-	*0.02*
Neurological Symptoms, no. (%)			
Cranial Nerve Involvement	16 (35.56%)	14 (48.27%)	0.27
Motor Deficit	23 (51.11%)	20 (68.96%)	0.13
Cerebellar Involvement	10 (22.22%)	4 (13.79%)	0.37
Babinski Sign	5 (11.11%)	4 (13.79%)	0.73
Coma	12 (26.67%)	7 (24.13%)	0.80
Sensory Deficit	9 (20%)	11 (37.93%)	0.09
Nuchal Rigidity	2 (4.44%)	4 (13.73%)	0.17
Epileptic Seizures	2 (4.44%)	-	0.43
Imagistic and Diagnostic, no. (%)			
Brain MRI	45 (100%)	28 (96.55%)	0.35
Spine MRI	11 (24.44%)	10 (34.48%)	0.35
Enhancing Lesions	19 (42.22%)	8 (27.58%)	0.20
AHLE	10 (22.22%)	3 (10.34%)	0.20
CSF-OCB	8 (17.78%)	4 (13.33%)	0.65
CSF-Anti-MOG	-	1 (3.44%)	0.35
Treatment, no. (%)			
Corticosteroids	37 (82.22%)	28 (96.55%)	0.09
IVIg	14 (31.11%)	10 (34.48%)	0.61
Rituximab	2 (4.44%)	2 (6.89%)	0.67
TPE	5 (11.11%)	8 (27.58%)	0.08
Corticosteroids + IVIg	13 (28.89%)	9 (31.03%)	0.66
Corticosteroids + Rituximab	2 (4.44%)	2 (6.89%)	0.67
Corticosteroids + TPE	4 (8.89%)	8 (27.58%)	*0.04*
IVIg + Rituximab	-	2 (6.89%)	0.18
IVIg + TPE	2 (4.44%)	3 (10.34%)	0.35
Rituximab + TPE	1 (2.22%)	1 (3.44%)	0.77
Corticosteroids + IVIg + Rituximab	-	2 (6.89%)	0.18
Corticosteroids + IVIg + TPE	2 (4.44%)	3 (10.34%)	0.35
IV-Ig + Rituximab + TPE	-	1 (3.44%)	0.35
All the Treatments	-	1 (3.44%)	0.35
Outcomes, no. (%)			
Full Recovery	3 (6.67%)	6 (20.68%)	0.09
Minor Sequelae	15 (33.33%)	17 (58.62%)	*0.02*
Major Sequelae	21 (46.67%)	3 (10.34%)	*0.002*
Death	5 (11.11%)	3 (10.34%)	0.87

The italic shows the significant values.

**Table 4:** Correlation between therapy options, coma, and AHLE and full recovery.

		Full Recovery All Patients	Full Recovery Postinfection	Full Recovery Postvaccination
Corticosteroid Monotherapy	Correlation Coefficient	0.384	0.274	0.529
	*p*-value	*0.001*	0.08	*0.003*
IV-Ig	Correlation Coefficient	*−0.277*	−0.202	−0.371
	*p*-value	*0.02*	0.20	*0.04*
Monotherapy	Correlation Coefficient	0.352	0.244	0.493
	*p*-value	*0.003*	0.11	*0.007*
Coma	Correlation Coefficient	−0.310	−0.318	−0.449
	*p*-value	*0.007*	*0.03*	*0.01*
AHLE	Correlation Coefficient	−0.06	0.07	0.665
	*p*-value	0.59	0.64	*<0.001*

The italic shows the significant values.

**Table 5:** Correlation between therapy options, coma, and AHLE and poor outcomes.

		Poor Outcome All Patients	Poor Outcome Postinfection	Poor Outcome Postvaccination
Corticosteroids	Correlation Coefficient	−0.337	−0.277	0.09
	*p*-value	*0.03*	0.08	0.61
TPE	Correlation Coefficient	0.382	0.314	0.256
	*p*-value	*0.01*	*0.04*	0.18
Corticosteroids + TPE	Correlation Coefficient	0.337	0.277	0.256
	*p*-value	*0.03*	0.08	0.18
Coma	Correlation Coefficient	0.501	0.389	0.449
	*p*-value	*<0.001*	*0.008*	*0.01*
AHLE	Correlation Coefficient	0.314	0.132	0.665
	*p*-value	*0.006*	0.38	*<0.001*

The italic shows the significant values.

## Data Availability

Data are available based on the request from corresponding author.
